# Study of the leaf anatomy in cross-section in the Iberian species of *Festuca* L. (Poaceae) and its systematic significance

**DOI:** 10.3897/phytokeys.83.13746

**Published:** 2017-07-14

**Authors:** Gloria Martínez-Sagarra, Pilar Abad, Juan Antonio Devesa

**Affiliations:** 1 Departamento de Botánica, Ecología y Fisiología Vegetal, Universidad de Córdoba, Edificio Celestino Mutis, Campus de Rabanales, 14071 Córdoba, Spain

**Keywords:** *Festuca*, Iberian Peninsula, leaf anatomy, sclerenchyma arrangement

## Abstract

A study of the leaf anatomy in the species of the genus *Festuca* present in the Iberian Peninsula was made. A total of 68 taxa were included and 15 characters were measured in leaf cross-section. The major anatomical features of each taxonomic group were characterized, and some variability was observed in the taxa. The anatomical patterns observed were compared and discussed with the relationships suggested by the molecular analyses. The leaf outline, the presence or absence of complete girders, and the development degree of the bulliform cells were the main characters to differentiate among fescue species of the fine-leaved clade and those of the broad-leaved clade. The most useful character to segregate species groups within the different taxonomic sections was the arrangement of the sclerenchyma, and a remarkable variability of this character was found in the species of Festuca section Festuca, especially in those located in other lineages according to molecular markers. Most of the anatomical patterns were not exclusive of the sections or lineages, and only some taxa could be anatomically differentiated at species level based on a set of non-taxative characters. The discordant pattern observed in *F. henriquesii*, a species traditionally included in Festuca sect. Festuca that shared anatomical features with the species of “*F. rubra* complex”, suggests its possible inclusion in the sect. Aulaxyper pending further taxonomic and phylogenetic analyses.

## Introduction

*Festuca* L. is one of the largest genera within the family Poaceae with more than 450 species mostly distributed in the temperate and alpine zones of both hemispheres (Watson and Dallwitz 1992; Clayton et al. 2006 onwards). Some fescue species are economically important worldwide for their forage value (e.g., *Festuca arundinacea* Schreb.), as well as for their use in turfs (e.g., *F. rubra* L.), gardening (e.g., *F. glauca* Vill.), and soil fixation (e.g., *F. ovina* L.).

The Iberian Peninsula has been considered one of the main speciation centres of the genus *Festuca* (Saint-Yves 1930), with about 100 taxa (between 70 and 80 species) organized in ten sections and three subgenera (Cebolla and Rivas Ponce 2003a; Devesa et al. 2013). It comprises rhizomatous and cespitose perennial species, both diploid and polyploid (up to 12*x* = 84 chromosomes; Fuente et al. 2001; Loureiro et al. 2007), capable of growing in a wide variety of environments and habitats (Kerguélen and Plonka 1989). Many of them are endemic species adapted to high mountain conditions (e.g., *F. indigesta* Boiss. and *F. pseudeskia* Boiss.), but they also grow in wet pastures (many species of the “*F. rubra* complex”), river areas, and forest edges [e.g., *F. gigantea* (L.) Vill.], and on coastal rocky cliffs and fixed coastal dunes, being able to tolerate high environmental salt levels [e.g., *F. vasconcensis* (Markgr.-Dann.) Auquier & Kerguélen and *F. juncifolia* Chaub.].

The phylogenetic analyses based on nuclear and chloroplast markers suggest that *Festuca* is a paraphyletic genus which should include other genera that were previously treated independently, such as *Lolium* L. and *Vulpia* C.C. Gmel. among others (e.g., Charmet et al. 1997; Torrecilla and Catalán 2002; Catalán et al. 2004; Inda et al. 2008). The fescue species are subdivided into two well supported clades: the “broad-leaved” and the “fine-leaved”, named so for the leaf shape of the species included in them. In general terms, the broad-leaved fescues have flat leaves, convolute or inrolled vernation, and the fine-leaved fescues have conduplicate or infolded leaves, and acicular, setaceous, or filiform innovation leaf blades (Catalán et al. 2007), although there are several exceptions (Namaganda and Lye 2008).

In the Iberian territory, the broad-leaved clade comprises the sects. *Schedonorus* (P. Beauv.) W.D.J. Koch (4 species), and *Plantynia* (Dumort.) Tzvelev (1 species) from subgenus Schedonorus (P. Beauv.) Peterm., the sect. Phaeochloa Griseb. (2 species) from subgenus Drymanthele Krecz. & Bobrov, and the sects. *Subbulbosae* Nyman ex Hack. (3 species), *Scariosae* Hack. (1 species), *Pseudoscariosa* Krivot. (1 species), and *Lojaconoa* Catalán & Joch. Müll. (2 species) from subgenus Festuca. The fine-leaved clade includes the sects. *Eskia* Willk. (5 species), and the more recently diverged sects. *Festuca* (subsections *Festuca* and *Exaratae* St.-Yves; ca. 45 species) and *Aulaxyper* Dumort. (ca. 15 species), all of them belonging to the subgenus Festuca. According to the molecular data, some species conventionally classified within sects. *Festuca* and *Aulaxyper* fall outside the clades that include their respective type species (Catalán et al. 2007), but the interspecific relations within those clades are not resolved or are poorly supported (Torrecilla et al. 2004).

The taxonomy of this genus is very complex due to the great morphological similarity between species and the high degree of overlap in the ranges of variation. The shortage of diagnostic morphological characters has favoured the study of complementary characters in order to clarify the taxonomic relationships between species and allow their correct identification. Anatomical features of the leaf blades in cross-section and those related to the micro-morphology of epidermal surfaces have been the main supplementary tools to add to the morphological characters used to characterize *Festuca* (e.g., Metcalfe 1960; Ellis 1976, 1979, 1986; Namaganda et al. 2009) and other genera of difficult taxonomy within the Poaceae family (e.g., López and Devesa 1991; Pimentel and Sahuquillo 2003; Kuzmanović et al. 2009; Gennaro and Morrone 2010; Ortúñez and Fuente 2010; Ortúñez and Cano-Ruiz 2013). Since Hackel (1882), leaf anatomy has been considered of taxonomic interest in the genus, and characters such as the outline of the leaf cross-section, the arrangement of sclerenchyma in relation to the vascular bundles, and the number of ribs and furrows are used around the world in combination with other morphological characters (e.g., Saint-Yves 1909; Metcalfe 1960; Ellis 1976; Markgraf-Dannenberg 1980; Kerguélen and Plonka 1989; Portal 1996; Clayton and Renvoize 1986; Fuente and Ortúñez 1998). The leaf anatomy has been especially investigated within the fine-leaved fescues clade, with a more complex taxonomy than the broad-leaved clade, and many taxa have been described or segregated mainly based on those anatomical characters (Namaganda et al. 2009).

Despite the extensive use of the leaf anatomy in *Festuca* and the importance of its systematics, many studies have evaluated environmental influences on the anatomical characters. Several authors have pointed out its restricted taxonomic value in *Festuca* (Connor 1960; Kjellqvist 1961; Aiken et al. 1985; Aiken and Consaul 1995; Ramesar-Fortner et al. 1995) and other grasses (Ruiz-Téllez et al. 1998; Giełwanowska et al. 2005; Kuzmanović et al. 2012; Olsen et al. 2013) because some features may be affected by ecological factors and by phenotype plasticity. However, although the identification of *Festuca* species using only anatomical variables is complex, most authors agree that it would reduce the possibilities of error and improve the separation of several similar taxa which were indistinguishable based on the morphology of vegetative and reproductive organs (Aiken et al. 1985).

In the Iberian Peninsula, leaf anatomy studies have usually been partial, accompanying species descriptions or in the treatments of regional Floras, and generally corresponding to iconographic details and diagrams or drawings of leaf cross-sections (e.g., Aizpuru et al. 1997; Fuente et al. 1997; Bolòs and Vigo 2001; Catalán 2009). On the occasion of the taxonomic study of the genus *Festuca* in the framework of the *Flora iberica* Project (Castroviejo et al. 1986–2017), the cross-section leaf anatomy of most species currently recognized for this territory have been analysed. In this work, we aim at improving our anatomical knowledge about the genus, and compare the leaf anatomy patterns with the latest molecular phylogenies.

## Material and methods

We analysed leaf sections in cross view of 68 Iberian taxa belonging to the subgenera *Festuca*, *Drymanthele*, and *Schedonorus* of the genus *Festuca*. Exceptionally, apart from the Iberian material, material from the French Pyrenees and from Andorra was selected. The species included and their nomenclature followed Devesa et al. (2013). The identification of the specimens was performed according to local Floras and monographs (Markgraf-Dannenberg 1980; Kerguélen and Plonka 1989; Fuente and Ortúñez 1998). Anatomical observations were based mainly on herbarium specimens from ABH, BC, COFC, FCO, GDAC, HUAL, MA, MAF, MGC, JACA, SANT and SEV (acronyms according to Thiers 2017), and also from fresh material collected in the field during the years 2012–2016 (specimens deposited in the COFC herbarium). About 400 preparations were made (several per individual). The list of plants examined anatomically, localities, and authorship of the species are given in Suppl. material 1.

Free-hand cross-sections of the penultimate innovation leaf blades were made directly on fresh or dry material, and subsequently hydrated in water, following the framework proposed by López and Devesa (1991). The cross-sections were mounted in 50% lactic acid, which helped to clear the cells. Observations and measurements were taken using a Motic BA300 light microscope equipped with an ocular micrometer. Photographs of leaf cross-sections were obtained using a Moticam 2500 digital microscope camera, and edited with Motic Images Plus 2.0 software.

**Figure 1.**
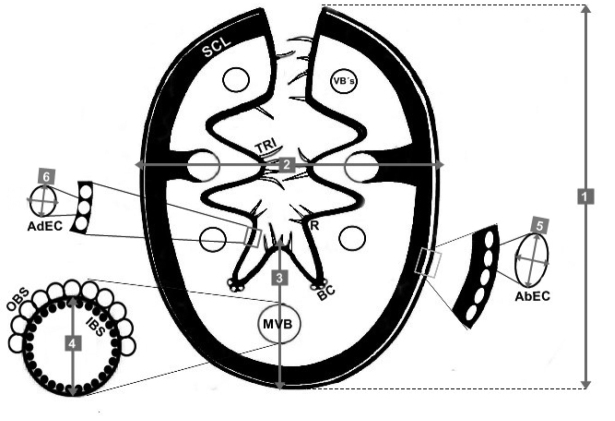
Main characters observed in the leaf cross-sections and abbreviations. Lines in grey indicate measures: **1** length (only in species with conduplicate blades) **2** maximum width **3** thickness of the blade at the midrib **4** maximum size/diameter of the median vascular bundle (**MVB**) **5** length × width of abaxial epidermal cells (**AbEC**) **6** length × width of adaxial epidermal cells (**AdEC**). Sclerenchyma (**SCL**); vascular bundles (**VB’sVB’s**); ribs (**R**); trichomes (**TRI**); outer bundle sheath cells (**OBS**); and inner bundle sheath cells (**IBS**).

**Figure 2.**
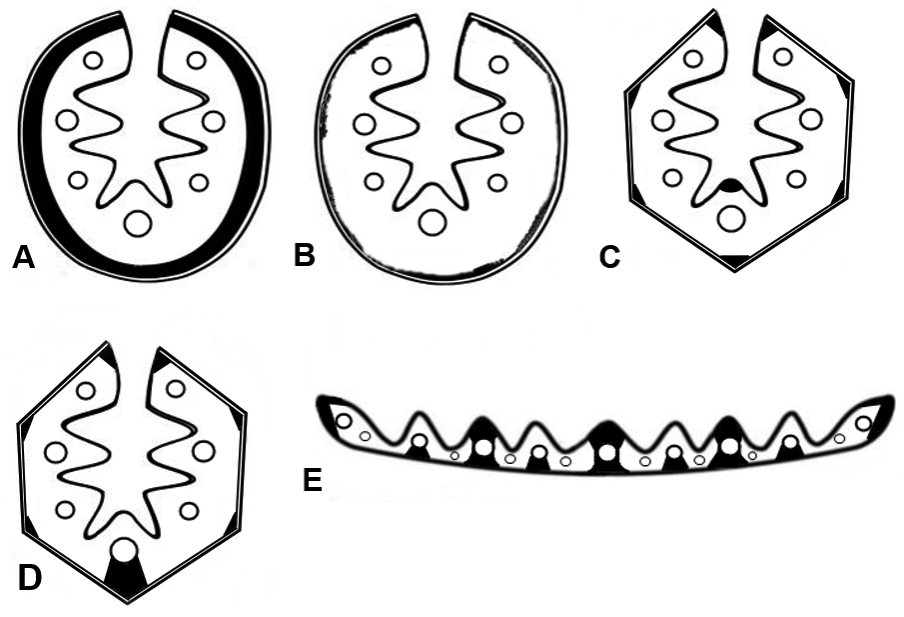
Major types of arrangement of sclerenchyma found in the leaf cross-sections. In conduplicate leaf blades: **A** continuous ring **B** interrupted continuous ring **C** forming abaxial strands and an adaxial strand on the median rib **D** with an abaxial girder at the median vascular bundle. In flat leaf blades: **E** complete (abaxial and adaxial) girder at the first vascular bundles, abaxial girder at the second vascular bundles, third vascular bundles without associated sclerenchyma.

The leaf anatomical characters observed were compiled from those mentioned in the literature on genus *Festuca*, as the outline, the pattern of abaxial and adaxial sclerenchyma arrangement, length, width (when the leaves are flat, the width was measured as the sum of the two hemilimbs), thickness at the midrib, number of vascular bundles, number of ribs, and number of bulliform cells (viewed in the grooves contiguous to the median rib). Other additional characters were added for anatomical characterization of the species, such as median vascular bundle diameter/maximum size, number of outer and inner bundle sheath cells, sclerenchyma thickness at the midrib, trichome density of the adaxial surface (glabrous, sparsely aculeate, or densely aculeate) and its length, and length and width of abaxial and adaxial epidermal cells (referred to the cell lumen from the lateral side). The terminology for the anatomical characters was based on Ellis (1976) and Metcalfe (1960). The main characters studied and positions of measurements made on each cross-section are illustrated in Fig. 1, and the main types of arrangement of sclerenchyma in Fig. 2.

## Results and discussion

**Figure 3.**
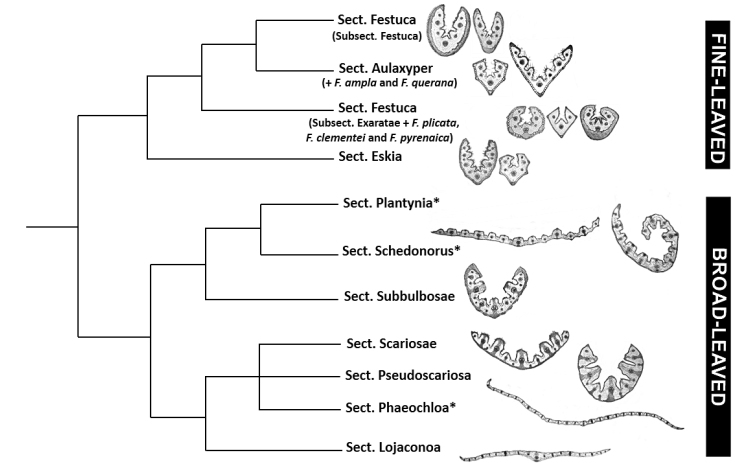
Simplified cladogram showing the major supraspecific relations in *Festuca* s.str., based and adapted on phylogenetic trees from Catalán et al. (2004), and Inda et al. (2008). Main leaf anatomical patterns are exemplified in each line evolutive. Abbreviations: L1, lineage 1; L2, lineage 2; and L3, lineage 3. Asterisks indicate sections which have been included in other subgenera by several authors (sect. Phaeochloa in the subgenus Drymanthele, and sects. *Schedonorus* and Plantynia in the subgenus Schedonorus).

**Figure 4.**
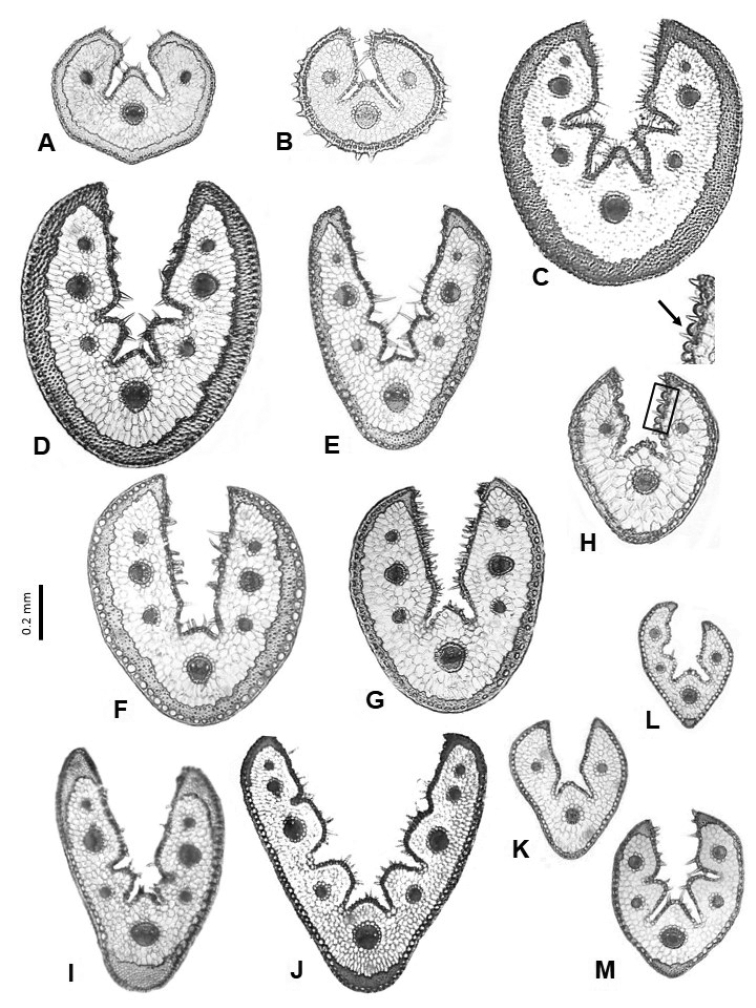
Leaf cross-sections of the Festuca sect. Festuca species from lineage 1. **A** *F. hystrix* **B** *F. reverchonii* **C** *F. segimonensis* **D** *F. indigesta* **E** *F. michaelis* **F** *F. glauca* **G** *F. vasconcensis* **H** F. brigantina subsp. actiophyta (the arrow indicates inflated adaxial epidermal cells) **I** F. marginata subsp. andres-molinae **J** *F. rivas-martinezii* **K** *F. frigida* **L** *F. alpina* **M** *F. glacialis*. Scale bars: 0.2 mm.

**Figure 5.**
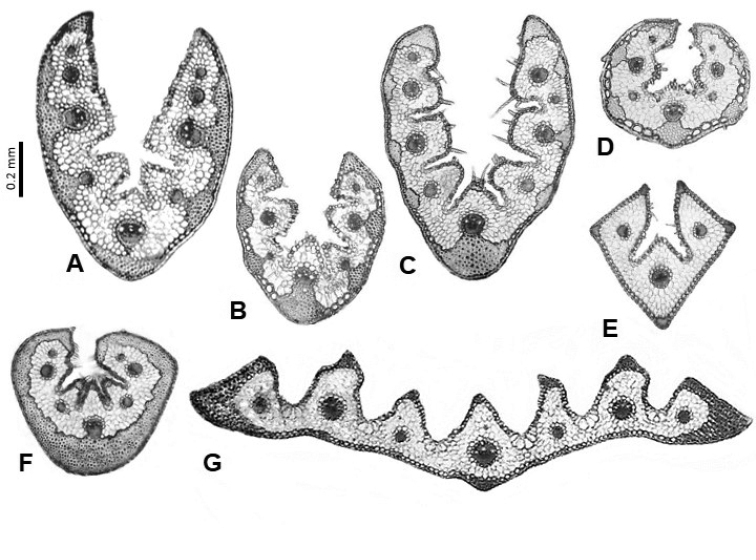
Leaf cross-sections of the Festuca sect. Festuca species from lineage 2: **A** *F. querana* **B** *F. ampla*; from lineage 3: **C** *F. borderei* **D** *F. capillifolia* **E** *F. plicata* **F** *F. clementei*; and unknown: **G** *F. henriquesii*. Scale bars: 0.2 mm.

**Figure 6.**
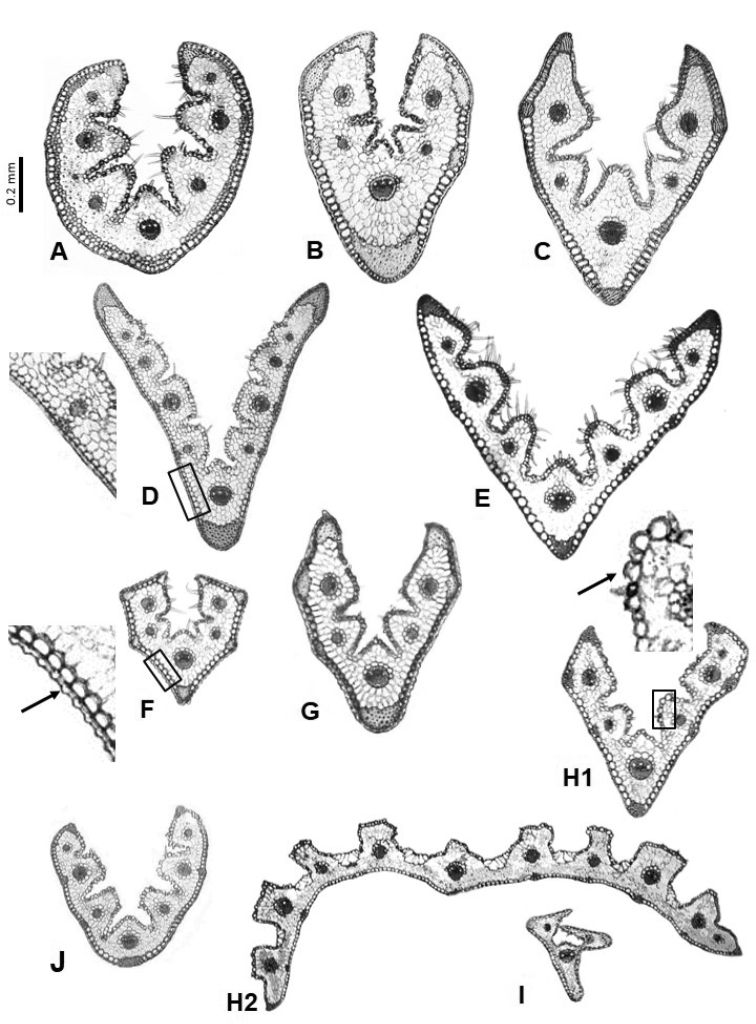
Leaf cross-sections of Iberian species of the Festuca sect. Aulaxyper from lineage 2: **A** *F. juncifolia* **B** F. rubra subsp. pruinosa **C** *F. rivularis* **D** *F. rothmaleri* (in detail abaxial surface not scalloped) **E** *F. nevadensis* **F** *F. iberica* (in detail scalloped abaxial surface) **G** *F. nigrescens* **H** F. heterophylla subsp. braun-blanquetii (H1 innovation leaf, in detail inflated adaxial epidermal cells; H2 cauline leaf) **I** F. heterophylla subsp. heterophylla (innovation leaf); and from lineage 3: **J** *F. pyrenaica*. Scale bars: 0.2 mm.

**Figure 7.**
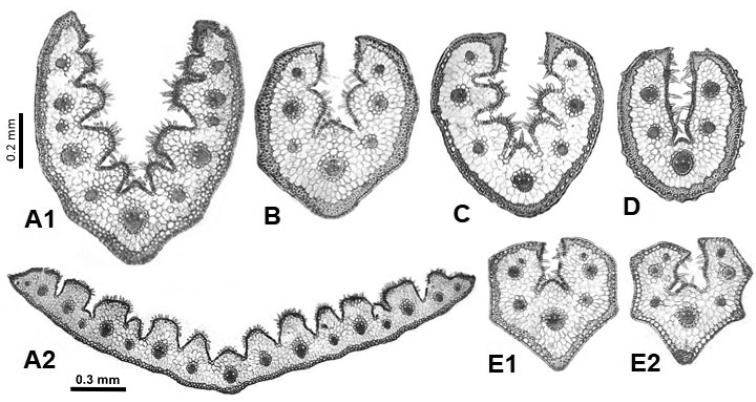
Leaf cross-sections of the Festuca sect. Eskia species. **A** *F. eskia* (A1 conduplicate leaf, A2 flat leaf) **B** *F. × picoeuropeana* **C** *F. burnatii* **D** *F. elegans* **E** *F. gautieri* (**E1** sclerenchyma in continuous ring, **E2** in strands). Scale bars: 0.2 mm (**A–E**), and 0.3 mm (**A2**).

**Figure 8.**
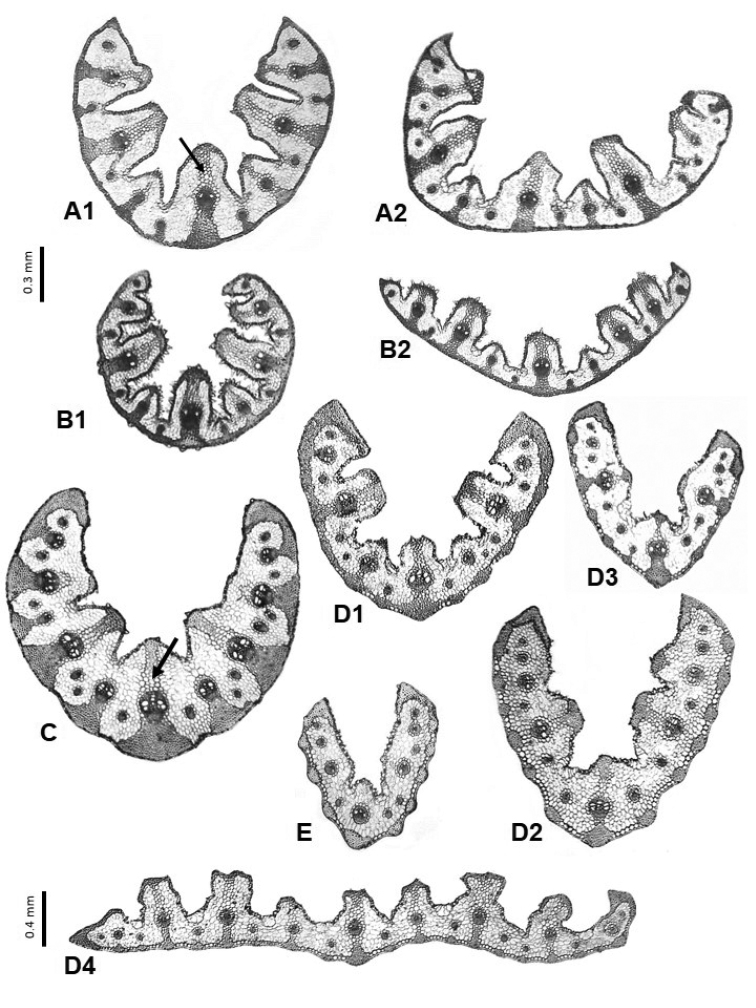
Leaf cross-sections of the *Festuca* sects. *Pseudoscariosa* (**A**), *Scariosae* (**B**) and *Subbulbosae* (**C–E**) species. **A** *F. pseudeskia* (**A1** conduplicate leaf, arrow pointing to the colourless cells; **A2** flat leaf) **B** *F. scariosa* (**B1** conduplicate leaf, **B2** flat leaf) **C** *F. baetica* (arrow pointing to the colourless cells) **D** *F. paniculata* s.l. (**D1** subsp. multispiculata, **D2** subsp. fontqueri, **D3** subsp. paui, **D4** subsp. longiglumis) **E** *F. durandoi*. Scale bars: 0.3 mm (**A–E**), and 0.4 mm (**D4**).

**Figure 9.**
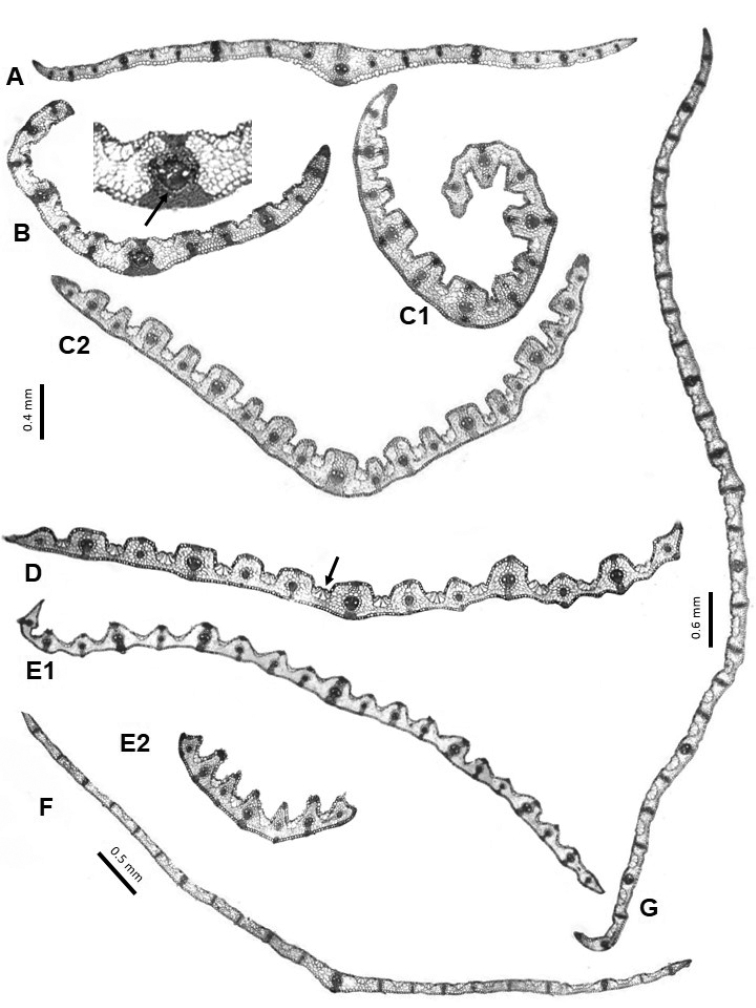
Leaf cross-sections of the *Festuca* sects. *Lojaconoa* (**A–B**), *Schedonorus* (**C–E**), and *Phaeochloa* (**F**–**G**) species. **A** *F. patula* **B** *F. coerulescens* (arrow pointing to the sclerenchyma interrupting the cells of the outer median vascular bundle sheath) **C** *F. interrupta* (**C1** inrolled leaf, **C2** flat leaf) **D** *F. arundinacea* (arrow pointing to the bulliform cells) **E** *F. mediterranea* (**E1** mature leaf, **E2** inmature leaf) **F** *F. altissima* **G** *F. lasto*. Scale bars: 0.4 mm (**A–E**), 0.5 mm (**F**), and 0.6 mm (**G**).

The studied anatomical characters in leaf cross view are summarized in Table 1. The species were grouped by subgenera and taxonomic sections according to traditional classification (Devesa et al. 2013), and subsequently were ordered by anatomical affinities. The lineage in which the fine-leaved species is located based on nuclear ITS and chloroplast *trn*L and *trn*L-F markers has been added when the molecular classification differs from the conventional one (Torrecilla and Catalán 2002; Torrecilla et al. 2004; Catalán et al. 2006, 2007; Nova et al. 2006; Inda et al. 2008). The main anatomical patterns found are discussed in relation to the two large clades recognized in molecular analyses within genus (*Festuca* s.str.) (Fig. 3): broad-leaved taxa and fine-leaved taxa (e.g., Charmet et al. 1997; Catalán et al. 2004; Inda et al. 2008). Illustrations of a selection of leaf cross-sections are provided in Figs 4–9.

**Table 1.** Leaf anatomical characters analysed in cross-section and its variation range in *Festuca* species. General abbreviations: **MVB**, median vascular bundle; **OBS**, cells of outer bundle sheath; **IBS**, cells of inner bundle sheath. Abbreviations in the clade or lineage: **Fl**, fine-leaved clade; **Bl**, broad-leaved clade; **L**, lineage (see Fig. 3). Abbreviations in the outline: **U**, U-shaped; **V**, V-shaped; **Y**, Y-shaped; **F**, flat; **r**, rounded; **e**, elliptical; **s**, straight; **a**, angled; **IL**, innovations leaves; **CL**, cauline leaves. Abbreviations in the sclerenchyma pattern: **C**, continuous; **Ci**, continuous ring sometimes laterally thickened, broken or scarcely interrupted, never in strands; **NP**, not present; **S**, strands, but not in contact with the bundle sheath; **G**, girders. Sub-indices indicate location of the girder: **MVB**, presence of girder of sclerenchyma only in central vascular bundle (in fine-leaved species); **LVB**, presence of girder of sclerenchyma only in lateral vascular bundles (in fine-leaved species); **1**, girder of sclerenchyma at first order vascular bundles; **2**, girder of sclerenchyma at second order vascular bundles; **3**, girder of sclerenchyma at third order vascular bundles (in broad-leaved species); * indicate girder-like extension of colourless cells near of the vascular bundles. Abbreviations in the OBS/IBS: **i**, OBS interrupted by sclerenchyma in the central vascular bundle; **nd**, no data. Abbreviations in the bulliform cells: **d**, developed; **ud**, undeveloped. Abbreviations in the trichomes of adaxial site section: **E**, densely aculeate; **A**, sparsely aculeate; **G**, glabrous. Numbers in square brackets indicates number of strands or strands plus girders. Letters and numbers in parentheses indicate uncommon values/character presence.

Taxa	Clade, lineage	Outline	Abaxial sclerenchyma pattern	Adaxial sclerenchyma pattern (in ribs)	Vascular bundles number	Ribs number	Length (mm)	Width (mm)	Thickness at the midrib (mm)	MVB max. size (µm)	OBS/ IBS number	Sclerenchyma thickness at the midrib (µm)	Bulliform cells number	Adaxial site trichomes (µm)	Abaxial epidermal cells, L × W (µm)	Adaxial epidermal cells, L × W (µm)
A. SUBGENUS FESTUCA																
*Sect. Festuca*																
*F. hystrix*	Fl	Ur	C	S[1]	3	1	0.44–0.60	0.52–0.70	0.33–0.44	70–87.5	12–16/ 19–22	37.5–75	3(5), ud	E, 12.5–37.5 (67.5)	7.5–20 × 5–10	7.5–12.5 × 5
*F. reverchonii*	Fl	Ur	C (Ci)	S[1]	3	1	0.32–0.46	0.30–0.46	0.19–0.25	70–77.5	13–15/ 20–25	10–20	5(6), ud	E, 15–37.5	7.5–12.5 × 5–7.5	7.5–12.5 × 5–10
*F. airoides*	Fl	Ur–e	C	NP or S[1]	5–7	1	0.54–0.68	0.37–0.52	0.24–0.30	67.50–80	10–13/ 21–23	12.5–50	3(4), ud	E(A), 15–47.5	7.5–15 × 5–10	7.5–12.5 × 5–10
*F. niphobia*	Fl	Ue	C	NP or S[1]	(5)7	1	0.56–0.75	0.43–0.50	0.27–0.32	67.5–85	12–13/ 19–23	27.5–42.5	3, ud	E, 17.5–50	7.5–12.5 × 7.5–12.5	7.5–12.5 × 2.5–7.5
F. brigantina subsp. brigantina	Fl	Ue	C	NP	5–7	1	0.60–0.91	0.44–0.69	0.31–0.39	72.5–100	8–15/ 18–24	22.5–67.5	4–5, ud	E, 10–87.5	10–27.5 × 7.5–15	7.5–17.5 × 5–10
*F. gracilior*	Fl	Ue–r	C (Ci)	NP	7	1–3	0.52–0.73 (0.80)	0.32–0.54	0.19–0.28	62.5–85	11–13/ 19–23	27.5–60	3–5, ud	E, 10–50	12.5–20 × 7.5–12.5	5–12.5 × 5–7.5
*F. michaelis*	Fl	Vs or Ue	C (Ci)	NP	7	(1)3	0.64–0.93	0.49–0.65	0.28–0.36	77.5–112.5	9–12/ 19–24	12.5–52.5	3, ud	E, 15–75 (112.5)	12.5–25 × 7.5–17.5	7.5–15 × 5–10
*F. valentina*	Fl	Ue–r	C	NP or S[1–3]	5–7	3(5)	0.72–0.80 (1.05)	0.51–0.74	0.30–0.47	82.5–112.5	9–14/ 19–25	50–82.5	3–4(5), ud	E, 15–95	7.5–27.5 × 5–15	7.5–15 × 5–10
*F. ochroleuca*	Fl	Vs or Ue	C (Ci)	NP	7(9)	1–3	0.54–0.86	0.42–0.69	0.20–0.33	65–107.5	7–13/ 16–27	15–55	3–4, ud	E, 22.5–40	12.5–20 × 7.5–12.5	7.5–12.5 × 5–10
*F. longiauriculata*	Fl	Ue–r	C	NP or S[1–3]	7	1–3	0.51–0.84 (0.90)	0.50–0.68 (0.81)	0.30–0.41 (0.43)	77.5–87.5	11–17/ 20–24	42.5–82.5	3–5, ud	E, 25–87.5	5–12.5 × 7.5–12.5	7.5–12.5 × 2.5–7.5
*F. vettonica*	Fl	Ue	C	S[3] (NP)	7	3	0.73–0.84	0.58–0.69	0.31–0.36	75.0–82.5	11–12/ 20–24	37.5–67.5	3, ud	E, 25.0–75.0	7.5–17.5 × 7.5–15	12.5–20 × 5–7.5
*F. aragonensis*	Fl	Ue	C (Ci)	NP or S[1]	5–7	1	0.52–0.73 (0.81)	(0.34) 0.44–0.50	0.25–0.36	65–100	9–16/ 19–24	20–57.5	2–3(4), ud	E, 12.5–75	12.5–20 × 7.5–17.5	10–15 × 7.5–10
*F. carpetana*	Fl	Ue	C	NP or S[1–2]	7(8)	1–3(5)	(0.60) 0.69–1.17	(0.50) 0.61–0.89	0.31–0.43 (0.50)	70–115	8–13/ 19–27	25–70	3–6, ud	E, 25–100	7.5–15 × 7.5–12.5	7.5–15 × 5–10
*F. summilusitana*	Fl	Ue	C	NP or S[1–3]	(5)6–9 (10)	1–3(5)	0.66–1.20 (1.41)	0.54–0.88	0.26–0.55 (0.60)	70–152.5	7–17/ 21–28	12.5–97.5	3–5, ud	E, 27.5–122.5	7.5–22.5 × 7.5–20	7.5–15 × 5–12.5
*F. gredensis*	Fl	Ue	C	NP or S[1–3]	7–9	1–3(5)	0.67–1.34	(0.53) 0.57– 0.94	0.32–0.49 (0.53)	85.0–155.0	9–14/ 19–24	20–50	3–5, ud	E, 15–112.5 (125.0)	10–25 × 7.5–20	10–17.5 × 5–10
*F. altopyrenaica*	Fl	Ue or Vs	C (Ci)	NP	7	1–3	0.90–1.15	0.61–0.95	0.31–0.41	75–112.5	8–12/ 19–23	22.5–32.5	3, ud	E, 27.5–75 (150)	10–20 × 7.5–20	7.5–12.5 × (5)7.5–10
*F. yvesii*	Fl	Ue or Vs	C	NP	7–9	1–3	0.80–1.18	(0.57) 0.72–0.90	0.33–0.44 (0.50)	95–125	10–15/ 20–26	25–80	3–5, ud	E, 25–87.5	12.5–25 × 10–20	10–15 × 7.5–10
*F. indigesta*	Fl	Ue	C	NP or S[1–3]	7–9(11)	(1)3(5)	0.80–1.52	0.75–1.11	0.35–0.57	117.5–130	13–15/ 20–26	62.5–87.5	3–5, ud	E, 45–125 (158)	10–15 × 7.5–12.5	7.5–17.5 × 5–10
*F. segimonensis*	Fl	Ur	C	S[3]	5–7(9)	3	0.70–1.1 (1.3)	0.69–1.02	0.38–0.67	80–107 (125)	10–16/ 20–26	37.5–50	4–6, ud	E, 32.5–100	12.5–17.5 × 7.5–12.5	12.5–20 × 5–7.5
*F. clementei*	Fl, L3	Ur–s	C, Ci, S [(3)5–7] or S+G_MVB_	S[1–3]	7	3(4)	0.45–0.58	0.52–0.66	0.28–0.38	62.5–72.5	11–14/ 19–22	75–175	3–7, ud	E, 10–55	7.5–15 × 5–12.5	5–12.5 × 5–15
*F. liviensis*	Fl	Ue	C (Ci)	NP	(8)9	3	(0.87)0.91–1.06	0.63–0.89	0.31–0.34	95–117.5	11–14/ 27–30	30–60	3–6, ud	E, 12.5–82.5	12.5–25 × 7.5–15	7.5–15 × 5–10
*F. glauca*	Fl	Ue	C (Ci)	NP	7(10)	1–3	0.79–1.15	0.55–0.76	0.30–0.36	85–107.5	11–12/ 24	40–50	3–4, ud	E, 50–125	17.5–37.5 × 15–32.5	7.5–17.5 × 5–12.5
*F. vasconcensis*	Fl	Ue–r	C	NP	5–7	1	0.75–0.97	0.66–0.85	0.35–0.45	85–115	8–13/ 19–25	15–30	3–5, ud	E, 10–55	15–37.5 × 10–25	10–20 × 5–17.5
F. brigantina subsp. actiophyta	Fl	Ue–r	Ci or S[3–6]	NP	3–5	1	0.46–0.62 (0.94)	0.40–0.61 (0.87)	0.26–0.35 (0.48)	65–90 (100)	6–9/ 16–20	12.5–25	4–6, ud	E, 15–37.5	15–35 × 10–25	12.5–25 × 7.5–20
F. marginata subsp. alopecuroides	Fl	Ue or Vs	S[3] (C)	NP	7	3	0.75–0.90	0.47–0.62	0.29–0.40	80–117.5	10–15/ 21–27	10–100	3–4, ud	E, 25–47.5	10–25 × 7.5–15	7.5–15 × 5–15
*F. rivas-martinezii*	Fl	Ue or Vs	S[3] (C)	NP	7(9)	3	0.70–1.02	0.60–0.90	0.25–0.44	85–117.5	11–22/ 21–33 (37)	35–60(95)	3–5, ud	E, (10)15–115	15–22.5 × 7.5–20	5–12.5 × 5–10
F. marginata subsp. andres-molinae	Fl	Ue Vs or Y	S[3] (C)	NP	7	(1)3	0.50–0.85	0.33–0.56	0.22–0.36	70–95	10–11/ 20–27	40–85	(1)3, ud	E, 10–60	15–22.5 × 7.5–10	5–12.5 × 7.5–10
F. marginata subsp. marginata	Fl	Ue, Vs or Y	S	NP	7(9)	(1)3	0.60–1.05	0.37–0.63	0.27–0.39	77.5–112.5	13–14/ 22–30	37.5–112.5	3–4, ud	E, (15)20–42.5	15–25 × 7.5–15	5–12.5 × 5–7.5
*F. frigida*	Fl	Ue or V	S[3]	NP	3	1	0.40–0.54	0.30–0.46	0.20–0.29	52.5–62.5	9–10/ 18–20	12.5–17.5	2–4, ud	A, 25–37.5	10–20 × 7.5–12.5	7.5–12.5 × 5–12.5
*F. alpina*	Fl	Ue or Vs_(a)_	S[3]	NP	3–4	1–3	0.45	0.36	0.20	57.5	12/ 17–20	30	4–5, ud	A, 25–37.5	15–20 (22.5) × 10–15	7.5–12.5 × 7.5–12.5
*F. glacialis*	Fl	Ue	S[3–5(7)]	NP	3–5	1–3	0.34–0.62	(0.24) 0.32–0.46	0.16–0.28	52.5–82.5	9–13/ 14–22	15–25	4–6, ud	E, 10–72.5	15–22.5 × 7.5–15	7.5–12.5 × 5–10
*F. plicata*	Fl, L3	Va	S or S+G_MVB_ [5(6)]	NP or S[1]	3(4)	1(2)	0.47–0.54	0.40–0.51	0.29–0.36	57.5–67.5	8–10/ 16–20	25–107.5	3–4, ud	A, 17.5–58	15–27.5 × 7.5–12.5 (20)	7.5–20 × 5–12.5
*F. capillifolia*	Fl, L3	Ua	S+G_MVB_ (S) [7(9)]	NP or S[1–3]	5–7	1–3	0.35–0.61	0.43–0.59	0.19–0.30	67.5–75	10–13 (16)/ 15–23	25–142.5	3, ud	E, 50–77 (110)	22.5–30 × 12.5–32.5	7.5–10 × 5–12.5
*F. ampla*	Fl, L2	Ue–r or Ua	G_VB_[7–9]	S[3–5]	(6)7(8)	(4)5	(0.44)0.52–0.78	0.50–0.8 (0.9)	0.21–0.32 (0.34)	72.5–112.5	9–12 (16)/ 17–23	45–120	3–6, ud	E(A), 17.5–55	12.5–32.5 × 10–25	10–25 × 10–20
*F. querana*	Fl, L2	Ue or Vs	S+G_LVB_[5]	NP	7–9(10)	3	0.72–1.01	0.41–0.55	0.20–0.35	80–92.5	12–13/ 22–23	50–70	4–5, ud	E or A, 15–30	12.5–17.5 × 7.5–12.5	7.5–15 × 7.5–12.5
*F. borderei*	Fl, L3	Ue or Vs_(a)_	S+G_MVB_[7–9]	NP (S[1])	7–9	5	0.66–0.96	0.55–0.73	0.24–0.35	62.5–85	10–14/ 22–24	87.5–125	4–7, ud	E, 32.5–87.5	12.5–25 × 7.5–15 (22.5)	7.5–15 × 5–10
*F. henriquesii*	Fl	F(V)	S[(3)5–7(8)]	S[5–7]	7–9(15)	5–7(9)		2.0–3.36	0.31–0.44	100–120	14–16/ 21–29	32.5–37.5	6, d	G or A, 22.5–30	10–20(25) × 10–17.5	7.5–17.5 × 7.5–15 (17.5)
Sect. Aulaxyper																
*F. rubra s*ubsp. *rubra*	Fl	Vs	S[7(10)]	NP	5–7(8)	3–5(6)	0.55–1.25	0.40–0.75 (1.3)	0.25–0.42	67.5–102.5	9–10/ 18–20	15–72.5	4–6, ud	E, 22.5–55(105)	15–32.5 × 12.5–30	10–22.5 × 10–22.5 (27.5)
*F. rubra s*ubsp. *juncea*	Fl	Va	S[5–7]	NP or S[1]	(6)7	3–5	0.79–1.08	0.54–0.81	0.37–0.45	92.5–112.5	10–12/ 17–22	100–132.5	3–7, ud	A or E, 12.5–37.5	17.5–25 × 12.5–22.5	10–22.5 × 7.5–17.5
F. rubra subsp. pruinosa	Fl	Vs–a (U)	S[7]	NP	5(7)	3	(0.71) 0.78–1	0.58–0.75	0.35–0.56	77.5–105	8–12/ 18–25	37.5–125	4–5, ud	E, 15–37.5	17.5–35 × 17.5–37.5	7.5–17.5 × 5–17.5
*F. iberica*	Fl	Vs	S[5–7]	NP	(3)5(7)	(1)3	0.36–0.63 (0.71)	0.33–0.52	0.19–0.34	62.5–85	9–12/ 17–22	30–50(100)	3–5, ud	E, (10)20–75(125)	17.5–27.5 × 10–22.5	10–17.5 × 7.5–20
*F. trichophylla*	Fl	Vs	S[(6)7]	NP	(4)5	1–3	0.47–0.57	0.33–0.49	0.24–0.30	57.5–90	9–12/ 15–19	50–62.5	3–5, ud	E, 25–62.5(107.5)	17.5–32.5 × 12.5–35 (42.5)	7.5–17.5 × 7.5–15
*F. rivularis*	Fl	Vs	S[5–7(8)]	NP	5–7	3(5)	0.61–1.06	0.55–0.90	0.29–0.50	85–92.5	9–12/ 17–21	14–20	3–5, ud	E, (18)25–100(120)	15–30 × 12.5–25 (37.5)	10–25 × 7.5–30
*F. nigrescens*	Fl	Vs	S[(6)7]	NP	5(6)	3	(0.42)0.59–0.80	0.38–0.60	0.20–0.35	62.5–77.5	8–9/ 17–19	35–77.5	4–5, ud	E(A), (10)20–37.5	15–30 × 12.5–20	7.5–17.5 × 7.5–20
*F. nevadensis*	Fl	V	S[(5)7–9]	S[5–7]	7(9)	5–7(8)	(0.65)0.75–1.30	0.74–1.25	0.23–0.45	82.5–120	9–13/ 16–21	50–120	3–5, ud	E, (20)42.5–125 (158)	20–40 × 12.5–37.5	12.5–22.5 × 7.5–15
*F. rothmaleri*	Fl	V	S[8–10]	NP	7–9	5–7	(0.56)0.79–1.07	0.41–0.85	(0.22)0.33–0.37	80–95	9–11/ 18–22	50–100	6–7, ud	E, (13)25–75(113)	12.5–30 × 12.5–27.5	7.5–17.5 × 7.5–22.5
*F. pyrenaica*	Fl, L3	Vs	S[9(11)]	NP or S[3]	7(9)	5(7)	0.57–0.92	0.52–0.61	0.17–0.25	57.5–75	9–10/ 15–21	22.5–25	3–4, ud	E, 15–37.5	12.5–20 × 10–20	7.5–15 (17.5) × 5–15
*F. juncifolia*	Fl	Ur–a	C or S[(6)7] (G_MVB_)	S[3–5]	7	3–5	0.67–0.98 (1.55)	0.61–0.86	0.31–0.39 (0.47)	85–100	9–12/ 18–22	47.5–52.5	3–5, ud	E, 12.5–85	12.5–30 × 17.5–32.5	7.5–17.5 × 5–17.5
F. heterophylla subsp. heterophylla	Fl	IL. Va	S[5]	NP	3	1	0.36–0.39	0.34–0.41	0.23–0.26	47.5–67.5	nd/ 14–18	15–27.5	4–5, ud	A, 17.5–37.5	12.5–5 × 5–12.5	7.5–15 × 5–10
		CL. F	S[9]	NP	7	5		2.32	0.16	85	nd/ 18	35–50	4–5, d	E, 10–102.5	12.5–30 × 10–20	10–15 × 10–12.5 (20)
F. heterophylla subsp. braun-blanquetii	Fl	IL. Va	S[(6)7]	NP	5–7	3	0.64–0.68	0.53–0.61	0.27–0.31	75–82.5	8–10/ 19–21	32.5–37.5	3–5, ud	A, 10–25	12.5–25 × 10–17.5	7.5–15 × 7.5–15
		CL. F	S[9]	NP	10	9–10		3.43	0.33	92.5	9/ 20	22.5	4–5, d	A, 12.5–14 (G)	17.5–27.5 × 12.5–15	7.5–20 × 5–20
Sect. Eskia																
*F. eskia*	Fl	Ue (F)	C	S[(6)7–13]	10–13 (17)	7–11 (13)	(0.75)0.89–1.10 (1.47)	0.72–1.02 (3.23)	0.27–0.38	80–102.5	10–13/ 17–22	30–75	3–5, ud	E, (13)20–65 (70)	7.5–20 × 5–12.5	7.5–12.5 × 5–10
*F. × picoeuropeana*	Fl	Ue–a	C	S[3–5(7)]	7(9)	3–5(7)	0.57–0.87	0.55–0.73	0.25–0.33	72.5–87.5	8–14/ 17–21	35–47.5	3–7, ud	E, 20–52.5	5–20 × 5–15	5–10 × 5–7.5
*F. burnatii*	Fl	Ue	C, Ci or S[7]	S[4–5(6)]	(5)7(9)	4–5(6)	(0.38)0.47–0.80 (0.90)	0.32–0.60	(0.19)0.26–0.32 (0.47)	57.5–8	7–10/ 13–16	20–62.5	3–5, ud	E, (17.5)22.5–57.5	17.5–30 × 10–17.5	7.5–12.5 × 7.5–10
*F. elegans*	Fl	Ue	C	NP	5	1	0.47–0.67 (0.70)	0.40–0.53	0.18–0.26 (0.30)	112.5–170	11–15/ 21–34	12.5–25	3–4, ud	E, (15)35–70	10–17.5 × 7.5–17.5	7.5–12.5 × 5–10
*F. gautieri*	Fl	Ua	C, Ci or S[7–9]	NP	5–7	1	0.44–0.67	0.53–0.60	0.28–0.46	72.5–92.5	9–14/ 17–24	25–75	4–7, ud	E, (10)20–42.5(47)	12.5–20 × 10–20	5–10 × 5–7.5
Sect. Subbulbosae																
*F. baetica*	Bl	Ur	(Ci or Ci+)G_1,2_ (S_3_)	G*_1_	17–23	3	1.03–1.66	0.99–1.64	0.49–0.74	142.5–200	18–25/ 30–36	105–232.5	4–7, d	E, 7.5–27.5(32)	10–22.5 × 5–10	7.5–15 × 5–15
*F. paniculata* *s.l.*	Bl	Ur (Vs or F)	(C or Ci+)G^(^*^)^_1_+S_2,3_	G*_1_(S_2,3_)	13–20	3–9	1.0–2.14	0.95–1.96 (4.18)	0.35–0.53	90–132.5	13–15/ 20–30	50–150	4–10, d or ud	A, (7)10–22(30)	15–32.5 × 7.5–25	7.5–17.5 × 7.5–17.5
*F. durandoi*	Bl	Ur or Vs	S[10–13]	NP or S[1–2]	11–13	1–3	0.66–0.89	0.66–0.95	0.29–0.36	75–92.5	11–12/ 19–22	47.5–62.5	4–7, d or ud	A, 7.5–27.5(G)	15–27.5 × 10–25	5–17.5 × 7.5–15
Sect. Lojaconoa																
*F. coerulescens*	Bl	F	G_1,2,3_	G_1,2,3_	10–14	8–12		1.67–2.25	0.20–0.26	90–110	i/ 18–22	27.5–42.5	4–7, d	G or A, 5–12.5	7.5–17.5 × 10–17.5	7.5–12.5 × 7.5–15
*F. patula*	Bl	F	G_1,2_+S_3_	G_1_+S_2,3_	12–17	11–16		(1.57)2.22–4.46	0.15–0.35	75–107.5	i/ 20–21	20–50	4–9, d	G	25–47.5 (57.5) × 17.5–42.5	10–20 × 7.5–17.5
Sect. Scariosae																
*F. scariosa*	Bl	Ur (F)	C(Ci)+G_1,2,3_	G*_1,2_	14–19	11–15	(0.74)0.89–1.67	0.97–2.39	0.37–0.54	117.5–132.5	14–16 (i)/ 26–30	75–112.5	4–7, d or ud	A(E), 20–37.5(38)	7.5–15 × 2.5–10	5–12.5 × 5–10
Sect. Pseudoscariosa																
*F. pseudeskia*	Bl	Ur (F)	G^(^*^)^_1,2_(G_3_)	G*_1,2_	15–18	5–8(9)	1.23–2.32	1.12–2.37 (2.72)	0.41–0.63	85–112.5	8–13/ 19–27	185–225	4–5, d or ud	E, 25–42.5	10–15 × 7.5–12.5	7.5–17.5 × 5–7.5
B. SUBGENUS DRYMANTHELE																
Sect. Phaeochloa																
*F. altissima*	Bl	F	G_1,2,3_	G_1,2,3_	25–32	23–30		5.44–10.16	0.12–0.27	87.5–92.5	i/ 18–22	22.5–47.5	5, d	G(A), 15–30	7.5–17.5 × 12.5–27.5	5–7.5 × 7.5–15
*F. lasto*	Bl	F	G_1,2,3_	G_1,2,3_	40–45	39–44		10.13–16.58 (20)	(0.20)0.26–0.38	105–137.5	i / 22–29	52.5–95	5–7, d	A, 7.5–45	10–15 × 10–22.5	7.5–15 × 10–17.5
C. SUBGENUS SCHEDONORUS																
Sect. Schedonorus																
*F. mediterranea*	Bl	F	G_1_+S_2_	S*_1,2_	7–20	(7)12–18		(1)1.24–7.09	0.23–0.34	82.5–110	11–17/ 18–25	57.5–80	(3)5–7, d	A, 15–37.5	10–15 × 20–25	12.5–22.5 × 17.5–25
*F. interrupta*	Bl	F	G_1,2_+S_3_	G*_1,2_	12–20	10–19		1.76–6.48	0.31–0.39	95–125	13–16/ 24–25	77.5–105	6–8, d	G or A, 30–42.5	10–15 × 12.5–22.5	12.5–22.5 × 12.5–27.5
*F. arundinacea*	Bl	F	G_1,2_(S_3_)	G*_1_+S_2_(S_3_)	13–17	11–15		4.30–12	0.23–0.28 (0.37)	95–130	12–15/ 22–24	62.5–75	5–9, d	G(A), 25–42.5	12.5–22.5 × 17.5–25	12.5–27.5 × 12.5–25
Sect. Plantynia																
*F. gigantea*	Bl	F	G_1,2,3_	G_1,2,3_	29–35	29–31		13–14.13	0.30–0.32	100–120	i/ 20–23	82.5–117.5	5–6, d	G(A), 22.5–30	12.5–30 × 17.5–30 (37.5)	12.5–20 × 12.5–30 (37.5)

### Leaf anatomy in the *Festuca* species of the fine-leaved clade

The fine-leaved fescues (see Fig. 3) present the outline of the blade in the transverse section usually conduplicate (from U- to V- or Y-shaped) and they usually do not exceed 1.5 mm in length, except for *F. henriquesii* and some rare forms of *F. eskia* with flat leaves (up to 3.3 mm wide). The sclerenchyma may be arranged in a continuous ring or forming strands that occasionally contact with the median or lateral vascular bundles, but never forming complete (abaxial and adaxial) sclerenchyma girders. The bulliform cells located in the intercostal zones are inconspicuous, and may even be absent (Table 1A, Figs 4–7).

This group comprises 3 taxonomic sections (sects. *Festuca*, *Aulaxyper*, and *Eskia*) which have been segregated into 4 different lineages according to the molecular phylogenies (e.g., Torrecilla et al. 2004; see Fig. 3). In those analyses, the location of some species from sects. *Festuca* (subsects. *Festuca* and *Exaratae*) and *Aulaxyper* differs slightly with the conventional classification (Fig. 3).

### Festuca sect. Festuca

The sect. Festuca (34 species analysed) in its traditional circumscription has the most diversity in anatomical features among the fine-leaved species. Three main patterns were observed regarding the arrangement of the sclerenchyma. The first pattern arranges sclerenchyma in a continuous ring, sometimes interrupted by just a few cells. The second pattern presents the sclerenchyma arranged in strands at the margins and midrib, rarely opposite the vascular bundles. The third pattern shows strands opposite the vascular bundles, frequently contacting the median or lateral vascular bundles (namely abaxial girder).

In the first pattern, the leaf is always conduplicate, often U-shaped and from elliptic to orbicular in outline, and may present from 3 to 9 (rarely 11) vascular bundles and from 1 to 3 (rarely 5) inconspicuous, slightly rounded ribs, with or without adaxial sclerenchyma strands (Table 1A, Fig. 4A–G). This is the most frequent anatomical model among the species of the sect. Festuca (subsect. Festuca) that dominate in high mountain pastures (about 21 species), although it is also present in the coastal taxa *F. vasconcensis*, *F. glauca*, and F. brigantina subsp. actiophyta (Table 1A).

Traditionally, the species of sect. Festuca with this anatomical pattern have been included within the broad “*F. ovina* complex”, which in turn includes groups of species with greater or lesser taxonomic difficulty (cf. Cebolla and Rivas Ponce 1999; Foggi et al. 2006; Pyke 2013; López et al. 2016). As in most species of the sect. Festuca, they have intravaginal innovations and sheaths usually open, as well as great morphological similarity. Almost all of these species grow in places that are dry, windswept, and nutrient deficient. They share a set of anatomical xeromorphic features such as strongly conduplicate leaves, of greater diameter in cross-section, highly developed sclerenchyma on the abaxial face, thick cell walls and abundant cutinization, small-sized lumen of the epidermal cells, bulliform cells barely visible or very small and relatively undifferentiated from the rest, and high density of trichomes on the adaxial face (Fig. 4). Many studies have indicated that the thickness of the sclerenchyma, and sometimes its arrangement and distribution, may vary depending on the environmental conditions, age and development of the leaf (Aiken and Consaul 1995). The sclerenchymatous protection may have played a major role in the survival of many grass species in such extreme ecological environments, since it confers mechanical support and protection, and contribute to rolling or folding of leaves, reducing water loss in drought conditions (Wrobel et al. 2007). The amount of basal sclerenchyma (maximum values of sclerenchyma found in *F. valentina*, *F. longiauriculata*, *F. indigesta*, and *F. summilusitana*), pubescence, and the size of the trichomes (maximum density and values in *F. summilusitana*, *F. gredensis*, *F. indigesta*, and *F. glauca*) are characters with high variability at intra- and inter-species levels, and could be a response to micro-environmental differences, therefore those have been considered just as descriptive characters (Connor 1960; Aiken and Consaul 1995; Ramesar-Fortner et al. 1995).

The length of the leaf section and the number of vascular bundles and ribs facilitated species distinction such as *F. hystrix* and *F. reverchonii*, the species of this group with the smallest diameters, characterized by having 3 vascular bundles and 1 median rib with sclerenchyma (Table 1A, Fig. 4A, B). These two species also present unique morphological characters within Festuca sect. Festuca such as the apex of the leaf being noticeably flattened in the first, and helicoid and scabrous leaves in the latter (Ortúñez et al. 1995). The remaining species show a considerable overlap of these characters which hampers the identification based solely on their leaf anatomy (Table 1A). For example, anatomy was useless to discriminate the species of the most complex groups within the sect. Festuca, as seen in the species studied of the “*F. inops* group” (*F. michaelis*, *F. inops*, and *F. valentina*), and for the species of the “*F. indigesta* group” (e.g., *F. indigesta*, *F. summilusitana*, *F. gredensis*, and *F. yvesii*, among others) (Table 1A). Within the latter group, only for *F. segimonensis* the leaf anatomy helped in its identification as the taxon has a more or less orbicular outline and presents adaxial sclerenchyma strands on its 3 ribs, which are more pronounced (with somewhat compressed bases) than in the other species (Fig. 4C).

Coastal species tend to have the largest abaxial epidermal cells (most visible in *F. glauca*; Fig. 4F), although with overlapping and not significant values. Generally, their subepidermal cells on the adaxial face are more or less inflated, which is a highly variable character even within the same population but that has been used by Fuente and Ortúñez (1998) to distinguish between *F. vasconcensis* (Fig. 4G) and other species. This character is also observed in F. brigantina subsp. actiophyta (absent in F. brigantina subsp. brigantina), another coastal taxon that was described from ultrabasic rocks of the northwest Iberian Peninsula (cf. Gutiérrez Villarías et al. 1997) and which is variable in the sclerenchyma arrangement (Table 1A, Fig. 4H). In this sense, it has been noted that, in addition to thick adaxial epidermis, plants growing near the sea present other adaptations against water loss under conditions of salt stress such as more-developed bulliform cells and atypical vascular bundles (Giełwanowska et al. 2005), although these characters were not observed in this study.

The molecular analyses group together all the species of sect. Festuca with continuous (or more or less interrupted) sclerenchyma within the first lineage of the fine-leaved clade (mostly subsect. Festuca species) (Fig. 3). Only *F. clementei* (Fig. 5F), a species that may have both continuous and discontinuous sclerenchyma, falls into lineage 3 together with the species of Festuca subsect. Exaratae and others close taxa (Fig. 3). Interestingly, in this species, the basal sclerenchyma is thicker and occasionally contacts the median vascular bundle (Table 1A), an anatomical feature that also appears in other phylogenetically related species (see below).

In the second pattern within sect. Festuca, the strands never make contact with the vascular bundles, and two variants can be recognized. The first variant is characterized by 3 strands of sclerenchyma, two marginal (apical in the cross-section) and one at midrib (basal in the cross-section), sometimes even visible externally on the leaf. The leaves are conduplicate, with elliptical cross-section, or in a V- or Y-shape, from 0.5 to 1 mm in length, with 7 (rarely 9) vascular bundles and 3 (more rarely 1) ribs without adaxial sclerenchyma strands (Table 1A). This pattern is present in F. marginata subsp. alopecuroides, F. marginata subsp. andres-*molinae*, F. marginata subsp. marginata, and *F. rivas*-*martinezii* which have traditionally been included in the “*F. marginata* group” (Fig. 4I, J). They are distributed over the centre, east, and northeast of the Iberian Peninsula, and some reach France. Some anatomical characters have been used in the taxonomy of this group such the ribs shape, which varies between rounded to more rarely truncate, and does not discriminate among these taxa, and the decurrence of the sclerenchyma strands (Kerguélen and Plonka 1989; Cebolla and Rivas Ponce 2003b). In the species studied, the arrangement of the marginal strands is very variable but that of the basal strand has taxonomic utility (although its observation requires some practice). It is decurrent in F. marginata subsp. alopecuroides and *F. rivas*-*martinezii* (Fig. 4J), and truncate and no decurrent in F. marginata subsp. marginata and F. marginata subsp. andres-*molinae* (Fig. 4I). Very rarely, the sclerenchyma was found to be continuous (Table 1A), making it very difficult in these cases to identify them from other “*F. ovina* complex” species. The species that share this anatomical pattern are included in lineage 1 of the fine-leaved clade (Festuca subsect. Festuca; Fig. 3). The AFLP and RADP markers seem to group these species together, although the *trn*L chloroplast marker does not discriminate affinities (Nova et al. 2006). In the second variant, the sclerenchyma may be arranged on the margins and the midrib, or also occasionally opposite some vascular bundles (Fig. 4K–M). The leaves are also conduplicate, but differ from the previous variant because the length of the leaf section is much smaller (0.34–0.62 mm), there are fewer vascular bundles (3 to 5), and the sclerenchyma strands are usually thinner, in addition to the adaxial trichomes being less abundant (Table 1A). This leaf model is that presented by the “*F. alpina* group” species which live in the alpine or subalpine habitats of the north (*F. alpina* and *F. glacialis*) and southeast (*F. frigida*) of the Iberian Peninsula. They are characterized by their small size, flexible leaves, and short panicles with few spikelets (López et al. 2016). The three species are phylogenetically very close (Catalán et al. 2007), and appear grouped within the lineage 1 (Festuca subsect. Festuca; Fig. 3).

In the third pattern, the sclerenchyma is opposite the vascular bundles, and frequently with an abaxial girder on the medial or lateral vascular bundle (Fig. 5A–E). This group includes species with anatomical characteristics intermediate between those of the previous species (lineage 1) and those of lineage 2 (mostly sect. Aulaxyper species, see below and Fig. 3). The majority of these species have a conduplicate leaf, and many of them are easily recognizable by their anatomy (Table 1A), an example being *F. plicata* (Fig. 5E) which has a trigonal or rhombic outline. *Festuca capillifolia* (Fig. 5D) presents an angular (polygonal) outline and differs from anatomically similar species such as *F. ampla* (Fig. 5B) because it does not present adaxial sclerenchyma on all of its ribs. *Festuca querana* (Fig. 5A) has a smooth outline and presents a more developed lateral abaxial sclerenchyma than the other species, even making contact with the vascular bundles, and neither does it present sclerenchyma on the ribs. The leaf anatomy of *F. borderei* (Fig. 5C) is similar to the previous species but it has a greater number of strands that in no case or very rarely contact the lateral vascular bundles and neither fuse abaxially. Interestingly, these anatomically heterogeneous species with unique anatomical patterns fall into a different clade from that formed by most species of sect. Festuca in the molecular trees (Catalán et al. 2004). Thus, *F. capillifolia* and *F. borderei*, which were already separated from the rest in the subsect. Exaratae due to morphological differences in their leaf sheaths (Saint-Yves 1922), are also differentiated in molecular phylogenies, being placed in a basal position (lineage 3) with respect to subsect. Festuca (lineage 1) and sect. Aulaxyper (lineage 2) (see Fig. 3). *Festuca plicata* seems to be more related to some species of the genus *Vulpia* and to the two previous species than to the rest of the species of subsect. Festuca (Catalán et al. 2004). Finally, *F. ampla* and *F. querana* are grouped together with the species of sect. Aulaxyper (Catalán et al. 2004; Nova et al. 2006), with which they share some anatomical characteristics.

Within this section, only one species, *F. henriquesii* (Table 1A, Fig. 5G), has a flat (or very broad V-shaped) leaf, with adaxial and abaxial strands on the margins and at the level of the vascular bundles which they do not make contact. Although there are no data on the phylogenetic relationships of this species, its leaf anatomy and some morphological characters such as closed leaf sheaths (Fuente and Ortúñez 1995) make it seem to be more related to the species of sect. Aulaxyper than those of sect. Festuca.

### Festuca sect. Aulaxyper

Almost all the species of this section (10 species analysed) share a pattern of abaxial sclerenchyma distribution of the leaves in strands opposite each of the vascular bundles, and at the leaf margins (Fig. 6). Although not very frequent, some species may present adaxial sclerenchyma on the ribs, which are well defined in this group. The leaves are frequently conduplicate, and exceptionally the cauline leaves may be flat (Table 1A). Only in *F. juncifolia* (Fig. 6A), a species that grows in the dunes and coastal sands in the north of the Iberian Peninsula, the abaxial strands frequently make contact forming a continuous or slightly disrupted ring. In most species, the leaf section has a polygonal outline (e.g., *F. iberica* and *F. nigrescens*; Fig. 6F, G), sometimes carinate (e.g., *F. rivularis* and *F. heterophylla*; Fig. 6C, H1, I), although with a great diversity of forms transitioning towards V-shaped (e.g., *F. rothmaleri* and *F. nevadensis*; Fig. 6D, E), and more rarely orbicular (*F. juncifolia*; Fig. 6A) or obovoid (some forms of F. rubra subsp. pruinosa, a species that is highly polymorphous in outline; Fig. 6B).

The only Iberian species of the genus with leaf dimorphism is *F. heterophylla* s.l., in which the cauline leaves are flat (Fig. 6H2) but those of the innovations are conduplicate and very narrow (especially in the subsp. heterophylla) (Fig. 6H1, I). In general, in the species of this section the abaxial epidermis cells are larger than those of the sect. Festuca (especially those in lineage 1), although variation in size was observed throughout the leaf cross-section, with greater values in the sides of the keel (remarkably in *F. rivularis*; Fig. 6). In addition, some of them are easily recognizable by their scalloped abaxial surface seen in cross-section (e.g., *F. iberica* and *F. trichophylla*; detail in Fig. 6F vs. not scalloped in Fig. 6D). In relation to the adaxial epidermis cells, it stands out that they are inflated in almost all the species of sect. Aulaxyper (Fig. 6, detail in Fig. 6H1), not only the coastal species as was the case in sect. Festuca. The thickness of the sclerenchyma strands, which has in some cases been used to separate some species (*F. nigrescens* vs. *F. iberica*), was found to be a very variable character, calling into question the taxonomic value that it had been given (Table 1A, Fig. 6F, G).

The species of sect. Aulaxyper (“*F. rubra* complex”) are characterized morphologically by their extravaginal innovations, with reddish-brown closed leaf sheath, generally fibrous (Hackel 1882). Most species live in woodland and meadows, on wet soils, and many are widely distributed throughout central, northern, and southwestern Europe (Markgraf-Dannenberg 1980). All the species of this group with the exception of *F. pyrenaica* (lineage 3) fall into lineage 2 (see Fig. 3), although, as also is the case in the sect. Festuca, the phylogenetic relationships between the species are not well defined. In *F. pyrenaica* the outline is elliptical or obovate, and neither does it present a girder in the median vascular bundle (Fig. 6J).

### Festuca sect. Eskia

The most frequent anatomical model for the species of this section is the sclerenchyma arranged in a continuous ring (Fig. 7A–E1), sometimes interrupted or forming irregular and decurrent strands as in *F. burnatii* (Fig. 7C), and only in *F. gautieri* might the sclerenchyma be continuous or arranged in well-defined or decurrent strands at the level of the vascular bundles (Fig. 7E2). The leaves are strongly conduplicate, although occasionally an individual of *F. eskia* was detected with more or less flat leaves (Fig. 7A2), both in the shoots from the sterile innovations and in the cauline leaves. This type of leaf was found in a plant that was growing in grassy and wet pastureland under the shelter of a rock, so it is possible that these variations are the result of phenotypic plasticity strongly influenced by environmental conditions. In fact, some *Festuca* species which have strongly folded leaves in dry places have been observed to have more or less flat leaves in moist conditions (Aiken et al. 1985).

In general, the sect. Eskia includes species with an outline that is elliptical (*F. elegans*; Fig. 7D), more or less obovate (*F. eskia* and *F. burnatii*; Fig. 7A1, C), sometimes slightly angular (F. × picoeuropeana; Fig. 7B), or markedly polygonal (*F. gautieri*; Fig. 7E1, E2), especially in those with sclerenchyma arranged in strands. In this sense, it has been shown that leaves with a more continuous sclerenchyma on the abaxial surface usually present a rounded and smooth outline, whereas those with sclerenchyma in strands have a more angular outline (Aiken and Consaul 1995). The largest leaf cross-section sizes are reached by *F. eskia* followed by F. × picoeuropeana and *F. burnatii*, while *F. gautieri* and *F. elegans* presented the smallest sizes (Table 1A). All of them have usually small-sized epidermal cells and abundant adaxial trichomes. They are glabrous on the abaxial surface, except *F. elegans* that is scabrous and also has a larger median vascular bundle diameter in proportion to the cross-section length (Fig. 7D, Table 1A). Section Eskia comprises species that share many anatomical features with the sect. Festuca but they are morphologically segregated from that section because they present broadly scarious lemmas and glumes and because of the shape of the ligule (Willkomm 1861; Fuente and Ortúñez 2001). The species of sect. Eskia inhabit alpine and subalpine pastures of the north of the Iberian Peninsula, being able to extend into the French Pyrenees, as well as in the mountains of the centre, northwest, and south of the Iberian Peninsula (Ortúñez and Fuente 2004; Torrecilla et al. 2013).

Anatomically, the *F. × picoeuropeana* hybrid shares intermediate anatomical characters with its parents, *F. eskia* and *F. gautieri*, mainly those referring to the outline, shape and number of ribs, and number of vascular bundles (Fig. 7B). Representatives from Festuca sect. Eskia were resolved as basal of the fine-leaved clade (Torrecilla et al. 2003, 2013).

### Leaf anatomy in the *Festuca* species of the broad-leaved clade

The broad-leaved taxa show two models of leaf cross-section: from narrowly flat (up to 2.4 mm wide, rarely reach 4 mm) to more generally conduplicate innovation leaf blades (up to 2.3 mm length) in the species of the sects. *Subbulbosae*, *Scariosae* and Pseudoscariosa (subgen. Festuca; Table 1A, Fig. 8); or completely flat (up to ca. 17 mm wide), with the leaves being more or less rolled up in prefoliation or under conditions of water stress (very visible especially in herbarium specimen sheets), as in the sects. Lojaconoa (subgen. Festuca; Table 1A, Fig. 9), Phaeochloa (subgen. Drymanthele; Table 1B), and sects. *Schedonorus* and Plantynia (subgen. Schedonorus; Table 1C).

Species of this clade present complete girders that extend from the vascular bundles to both the abaxial and the adaxial epidermis (except in *F. durandoi*), which can contact the abaxial face with a continuous or discontinuous ring. The girders may consist only of small sclerenchyma cells with thickened and lignified walls, sometimes interrupting the outer bundle sheath, or the outer sheath may possess girder-like extensions contacting with the sclerenchyma tissue, on both the abaxial and the adaxial faces. The density and size of the adaxial trichomes are less than in the fine-leaved fescues, particularly marked in the species with a completely flat leaf section where they may be glabrous or present small and very scattered aculei (Table 1). Noticeable in most species is the presence of large, highly developed bulliform cells in the intercostal spaces, making the leaf blade unfold (see exceptions in sects. *Subbulbosae*, *Scariosae* and *Pseudoscariosa*). In this great clade, 7 lineages are recognized that correspond to 7 well-defined taxonomic sections (Fig. 3), which have been distributed in three different subgenera.

### *Festuca* sects. *Scariosae* and *Pseudoscariosa*

The sects. *Scariosae* (*F. scariosa*) and *Pseudoscariosa* (*F. pseudeskia*) have very similar anatomical patterns (Fig. 3). The leaves are more or less conduplicate, although sometimes they can have very extended blades and be almost flat, with the midrib only slightly differentiated (Fig. 8). They are characterized by the presence of adaxial and/or abaxial sclerenchyma girders (usually T-shaped on the adaxial side) in the first-order and second-order vascular bundles, associated with extensions of the bundle sheath composed of large, thin-walled, colourless cells of the same size as or larger than the outer bundle sheath cells, and girders or strands facing the third-order vascular bundles only on the abaxial face, or sometimes without sclerenchyma. The girders may finish in a sclerenchyma ring as in *F. scariosa* (Fig. 8B), or not as in *F. pseudeskia* (Fig. 8A). In cross-section, both species present deep heteromorphic furrows, rounded or sometimes truncate or slightly triangular. Bulliform cells are less developed in the conduplicate forms of these sections (Fig. 8A1, B1), sometimes unnoticeable, whereas they are far more developed in the extended or more or less flat forms (Fig. 8A2, B2). Epidermal cells are small in both species, especially those on the abaxial face in *F. scariosa*, most likely due to the presence of the continuous ring and a strongly cuticularized epidermis. They grow in the south and southeast of the Iberian Peninsula, and present mixed shoots and short rhizomes (Fuente and Ortúñez 2001). Given their morphological characteristics, both taxonomic sections have been included within the broad subgenus Festuca, although phylogenetic reconstructions show that they are more related to species of the sect. Phaeochloa (e.g., Torrecilla et al. 2003; Fig. 3), with which, however, they show major anatomical differences (Fig. 9, see below).

### Festuca sect. Subbulbosae

The anatomical model of the species of the sect. Subbulbosae hardly differs from the previous ones, except that complete girders (T-shaped and usually with colourless cells towards the adaxial epidermis) are only found in the main vascular bundles and are usually absent in the secondary vascular bundles (Fig. 8C–E). Anatomical differences have been found between species with regard to the arrangement of the sclerenchyma and the form and number of ribs (Fig. 8C–E). Some variations of *F. paniculata* s.l. have been found and these may correspond to different infraspecific categories (Fig. 8D1–D4). One variant is characterized by a flat section, a great number of vascular bundles, with complete girders in the first- and second-order vascular bundles, and strands in those of third-order, which never end in a ring on the abaxial face, and that could correspond with F. paniculata subsp. longiglumis (Fig. 8D4), the only subspecies of the “*F. paniculata* group” for which completely flat leaves have been described. More frequent is the variant characterized by having conduplicate (U- or V-shaped) leaves, whose arrangement of the abaxial sclerenchyma may be in the form of a continuous band (F. paniculata subsp. multispiculata; Fig. 8D1), an almost continuous band with the base of the girders swollen (*F. baetica*; Fig. 8C), or in strands which confluent with the first-order vascular bundles (F. paniculata subsp. fontqueri and F. paniculata subsp. paui; Fig. 8D2, D3, respectively). *Festuca durandoi* (Fig. 8E) differs from all the foregoing species in that it presents neither complete sclerenchyma girders nor adaxial girders, and it have underdeveloped strands, generally smaller leaf length and width, and lateral ribs absent or inconspicuous.

The sect. Subbulbosae was also traditionally included in the subgenus Festuca, being characterized by the presence of intravaginal innovations, leaves with swollen bases that confer a sub-bulbous appearance, and split sheaths. However, phylogenetically it is located in the same clade as the sects. *Plantynia* and *Schedonorus* with which it has evident anatomical differences (Torrecilla and Catalán 2002).

### Festuca sect. Lojaconoa

The leaf model of sect. Lojaconoa (*F. patula* and *F. coerulescens*) shares many characteristics with the rest of the taxonomic sections with flat leaves (Fig. 3, Table 1B, C). *Festuca patula* (Fig. 9A) only presents girders in the primary and secondary vascular bundles, and has a greater number of vascular bundles. Its adaxial surface is almost smooth with the intercostal spaces defined only by the bulliform cells, and the midrib projects abaxially. *Festuca coerulescens* (Fig. 9B) presents complete girders in all its vascular bundles, and generally has fewer vascular bundles and ribs, the latter being truncate and with relatively pronounced intercostal spaces. In both, the sclerenchyma girders interrupt the outer first-order bundle sheath cells in the adaxial and abaxial surfaces, and are not associated with colourless cells. These may sometimes be present but only associated with lower order vascular bundles towards the adaxial face. The margins finish in two sclerenchyma strands, and never have a ring on the abaxial face. In addition, the epidermal cells are clearly visible on the abaxial face, which is glabrous or slightly aculeate. They are the only species of the subgenus Festuca with totally flat leaf blades, and are characterized morphologically by basally swollen leaf sheaths (Müller and Catalán 2006).

### Festuca sect. Phaeochloa

The anatomical pattern presented by the species of this section (*F. altissima* and *F. lasto*) is quite homogeneous (Fig. 9F, G), although with variability in width, and numbers of ribs and vascular bundles. In both, the sclerenchyma forms complete girders in all the vascular bundles and interrupts the outer bundle sheath cells in the adaxial and abaxial surfaces (Table 1B). They are two of the species with the greatest leaf widths of the entire genus (*F. lasto* is the largest of the genus with up to 16.6 mm, rarely 20 mm), and have practically smooth adaxial surfaces since the intercostal spaces defined by the bulliform cells are weakly or not at all developed. The bulliform cells are clearly visible, as in all the species with flat leaves of this clade. *Festuca lasto* grows in the south of the Iberian Peninsula, while *F. altissima* inhabits wet zones of the north of this territory, and extends over Europe (Devesa et al. 2013). Both species are elsewhere characterized morphologically by extravaginal innovations and absence of auricles (Hackel 1882; Clayton and Renvoize 1986), and are included in the subgenus Drymanthele.

### *Festuca* sects. *Plantynia* and *Schedonorus*

In the species of these sections, the leaves are flat, with more or less open or fully expanded hemilimbs (Table 1C, Fig. 9). Only *F. interrupta* may have convolute leaves more or less inrolling from one margin (Fig. 9C). No clear anatomical models associated with these taxonomic sections can be appreciated. The species of the sect. Schedonorus (*F. interrupta*, *F. arundinacea* and *F. mediterranea*; Fig. 9C–E) only have complete girders in the primary and secondary vascular bundles. In addition, the sclerenchyma makes contact with adaxial extensions of colourless cells in the median vascular bundle, sometimes also in the abaxial site (seen in *F. arundinacea*). The ribs are well defined in all of these species, from truncate to rounded in *F. arundinacea* (Fig. 9D) and *F. interrupta*, in which they are peculiarly heteromorphic in size and form (Fig. 9C), while in *F. mediterranea* they range from rounded to triangular (Fig. 9E). In *F. gigantea* (sect. Plantynia), the girders are complete in all the vascular bundles and the outer sheath is interrupted, and the ribs are rounded or truncate. Of these species, *F. arundinacea* and *F. gigantea* have the largest leaf widths (up to 12 mm and 14 mm, respectively), while *F. interrupta* and *F. mediterranea* have the smallest (up to 6.5 mm and 7.1 mm, respectively). The bulliform cells appear markedly larger and inflated, and arranged in a fan shape in the intercostal areas in all of these species. The adaxial surface may be glabrous or slightly aculeate. All these species have been integrated into the subgenus Schedonorus (Inda et al. 2014), and are characterized morphologically by having sterile extravaginal shoots with cataphylls and clasping falcate auricles.

### Conclusion and final remarks

Leaf anatomy as seen in cross-section has certain limitations for the delimitation of species, although it has taxonomic value for the separation of some groups. How useful anatomical characters is closely related to the taxonomic level that one wants to discriminate. Thus, the anatomical differences between the species of the two major clades are evident, and there are many features that distinguish them. Fine-leaved fescues usually present strongly folded leaves, rarely flat, with continuous sclerenchyma or strands, but never forming complete girders nor having colourless cells associated with the girders, and with bulliform cells that are relatively unpronounced. Fescues of the broad-leaved clade may present a leaf blade from convolute to fully folded, almost always with sclerenchyma girders associated with colourless cells, and highly developed bulliform cells.

Within the fine-leaved fescues clade, the character that most discriminates the taxonomic sections, the groups of species, and the species, is the arrangement of the sclerenchyma. Its analysis in species whose phylogenetic placement puts them in different lineages than what had been expected according to traditional taxonomy affects the previously recognized anatomical models, especially for the sects. *Festuca* and *Aulaxyper*. In species of the sect. Festuca included in lineage 1 (Fig. 3) and in the sect. Eskia, the leaves generally present smooth outlines, and there predominates a continuous or continuous-interrupted arrangement of the sclerenchyma, more rarely in strands in the margins and the midrib or opposite the vascular bundles. In contrast, in species of the sect. Festuca included in lineage 3 and lineage 2 (Fig. 3), the leaves have angular outlines, and there predominates discontinuous sclerenchyma opposite the vascular bundles.

The length and width of the leaf cross-section, and the number of vascular bundles and ribs overlap in most species of this clade, although they are useful for the differentiation of some taxa within the same taxonomic section and/or lineage. Only *F. henriquesii*, a species traditionally placed in the sect. Festuca, has a flat or a wide V-shaped leaf, and its pattern is very different from that typical of species of lineages 1 or 3 (Fig. 3). The arrangement of the sclerenchyma into strands without forming complete girders, the presence of developed ribs, and the number of vascular bundles suggest a greater affinity with the species of the sect. Aulaxyper, in which there are species whose leaf cross-sections present extended arms (*F. nevadensis* and *F. rothmaleri*) or are flat in their cauline leaves (*F. heterophylla*).

Species can neither be distinguished nor grouped together by the remaining characters studied, since they overlap to a great extent (especially in the number of outer/inner bundle sheaths cells, and the number of bulliform cells), and many of the variations found (e.g., thickness of the sclerenchyma, and abundance and length of the trichomes) may be responses to environmental conditions. The size of the lumen of the epidermal cells may be useful to differentiate certain species (*F. glauca*, *F. vasconcensis*, F. brigantina subsp. actiophyta, *F. iberica*, and *F. trichophylla*), although some heterogeneity was found. Also, a major intraspecific variability was found, especially in the sclerenchyma pattern and the degree of folding of the leaf, which is particularly striking in *F. eskia*.

In the species of the broad-leaved clade, some anatomical features are associated with the shape of the leaf, which may be conduplicate or totally flat. The variations observed affect the size, the presence of girders, their arrangement relative to the vascular bundles, the presence of colourless cells, and the development and shape of the ribs. Thus, the species of the sects. *Scariosae*, *Pseudoscariosa*, and *Subbulbosae* are anatomically the most similar, but they are very different from those of the sect. Lojaconoa which has been included within the same subgenus Festuca. All except those of the sect. Lojaconoa have a leaf pattern that varies from conduplicate to more or less flat, very evident ribs, T-shaped girders, and a major overlap in the numbers of vascular bundles and ribs. The main differences between them have to do with the arrangement of the sclerenchyma with respect to the vascular bundles, and the presence of a ring that may or may not be continuous. *Festuca durandoi* is the species that is anatomically farthest from the rest of this group, it being the only one that has no complete sclerenchyma girders. In the remaining sections of the broad-leaved clade, the species have flat leaves and greater leaf width and numbers of vascular bundles and ribs, and some of them may be recognized by the ribs being absent or poorly developed (*F. altissima*, *F. lasto*, and *F. patula*) or by whether or not the sclerenchyma contacts the outer bundle sheath towards the adaxial face.

The leaf anatomy has, on the one hand, a clear practical interest from an ecological and agronomic point of view for the early recognition (e.g., vegetative stages) of many species of *Festuca*. From a systematic view, anatomical patterns reinforce the morphological and molecular delimitation of some taxonomic sections or groups of taxa, although some of these patterns or models may appear in different sections or be very different in closely related sections. It would be interesting to assess anatomically all genera currently included in the genus *Festuca* s.l. (e.g., *Vulpia*, *Wangenheimia*, *Ctenopsis*, *Lolium* and *Castellia*, among others), whose leaf anatomy is less known because it is not a diagnostic character in their taxonomy, with the aim of exploring the global anatomical diversity patterns in the different lineages.

## Supplementary materials

Supplementary material 1 Additional information Link: https://doi.org/10.3897/phytokeys.83.13746.suppl1 Data type: species data Explanation note: List of taxa and localities of herbarium specimens used for the leaf cross-section anatomical study. Subgenera and taxonomic sections are arranged as in Table 1, and the taxa appear in alphabetical order within each section. This dataset is made available under the Open Database License (http://opendatacommons.org/licenses/odbl/1.0/). The Open Database License (ODbL) is a license agreement intended to allow users to freely share, modify, and use this Dataset while maintaining this same freedom for others, provided that the original source and author(s) are credited.Gloria Martínez-Sagarra, Pilar Abad, Juan Antonio Devesa
