# Supplementary material for: Study of the leaf anatomy in cross-section in the Iberian species of Festuca L. (Poaceae) and its systematic significance
Source: PhytoKeys. 2017 Jul 14;(83):43–74. doi: 10.3897/phytokeys.83.13746 (PMC5624202; doi:10.3897/phytokeys.83.13746)
Supplement: Supplementary material 1 — Additional information [file phytokeys-83-043-s001.pdf]

Appendix 1. List of taxa and localities of herbarium specimens used for the leaf cross-section anatomical study. Subgenera and taxonomic sections are arranged as in Table 1, and the taxa appear in alphabetical order within each section.

A. SUBG. FESTUCA

Sect. Festuca

Festuca airoides Lam. **FRANCE. Pyrénées-Orientales**: Perpiñán, Portè-Puymorens, Pic Carlit, 10 Aug 2011, J.V. Ferrández Palacio (JACA 288525).
SPAIN. Gerona: Vall de Nuria, road to Pico Noufont from the Sanctuary of Núria, 23 Jul 2014, E. López & G. Martínez (COFC 61416); Vallter 2000 Ski Resort, Plans de Coma Armada, 9 Jul 1999, R. Gamarra & E. Ortúñez (JACA 55599); **Lérida**: Collada Barradós, Valle de Arán, 9 Jul 1992, P. Montserrat, J.A. Sesé & J.L. Benito (JACA 232692); Puig de la Baqueira, Valle de Arán, 09 Jul 1966, S. Rivas-Martínez (JACA 309295).

Festuca alpina Suter. **SPAIN**. **Lérida**: Espot, Sota el Bony de las Picardes, 20 Aug 2012, Guardiola & Petit (BC 926491).

Festuca altopyrenaica Fuente & Ortúñez. **SPAIN**. **Huesca**: Aisa, border with Borau, Sayerri, 17 Jul 1985, P. Montserrat & D. Gómez (JACA 233685); Aso de Sobremonte, Peñas de Aso, 9 Aug 1984, P. Montserrat et al. (JACA 128684); Borau, Las Blancas, Lecherín Alto, 22 Jul 1967, P. Montserrat & F. González Bz. (JACA 588767); Jaca, Oroel, 25 Jun 1991, P. Montserrat (JACA 106591); Plan, Collado Coronas, 16 Aug 1980, P. Montserrat & L. Villar (JACA 396980).

Festuca ampla Hack. **PORTUGAL**. **Algarve**: Fóia, 18 May 2016, J.A. Devesa & G. Martínez (COFC 63232); between Fóia and Monchique, 18 May 2016, J.A. Devesa & G. Martínez (COFC 63233). **SPAIN**. **Guadalajara**: Luzaga, 26 May 2013, G. Martínez & C. Morales (COFC 61513); Ibídem, 22 Jun 2013, C. Morales (COFC 61269); **Huelva**: El Arrayán, 27 Apr 2016, J.A. Devesa & S. Talavera (COFC 63228); Hinojos, Cancela Cabeza Rasa, 27 Apr 2016, J.A. Devesa & S. Talavera (COFC 63229); Ibídem, Las Palomas, 27 Apr 2016, J.A. Devesa & S. Talavera (COFC 63226); Villablanca, 28 May 1984, J. Arroyo, J.A. Mejías & S. Talavera (MA 454062); **Madrid**: Pedrezuela, Guadalix river Valley, 24 May 2013, G. Martínez & C. Morales (COFC 61518); **Málaga**: Antequera, La Fresneda state, Co mountain range, Peña Negra, lane to Enebral farmhouse, B. Cabezudo & F. Soriguer (MGC 83322); Colmenar, Tajos de Marchena, between Sierra Prieta and Camarolos mountain range, 15 Jul 2016, B. Cabezudo & F. Soriguer (MGC 83983); Sierra de las Nieves Natural Park, Los Quejigales, 28 Jun 2012, B. Cabezudo, R. Casimiro-Soriguer, J.A. Devesa & E. Ruiz de Clavijo (COFC 61015); **Segovia**: Villar de Sobrepeña, Duratón river canyon, 3 Jun 1982, C. Cebolla, M.A. Rivas & C. Soriano (MA 366378).

Festuca aragonensis (Willk.) Fuente & Ortuñez. **SPAIN**. **Soria**: Ágreda, Moncayo, pass between the top of Santuario and Moncayo peak, 27 Jun 1995, E. Ortúñez & L.M. Ferrero (MAF 159615); **Zaragoza**: Añón, Circo de Morca, 7 Jul 1999, D. Gómez & P.M. Uribe-Echebarría (JACA 507399); Tarazona, ascension to Moncayo from the Santuario, 25 May 2014, E. López & G. Martínez (COFC 61546); Ibídem, Moncayo, 27 Jun 1995, E. Ortúñez & L.M. Ferrero (MAF 159613 & MAF 159614); Ibídem, Pico

Nariz, 20 Jun 2000, P.M. Uribe-Echebarría (JACA 48600); Ibídem, San Miguel glacier circus or of the Cucharón, 10 Jun 1999 (JACA 261412).

Festuca borderei (Hack.) Hack. in K. Richt. **FRANCE**. **Pyrénées-Orientales**: Err, Puigmal-N, 5 Jul 1986, P. Montserrat, L. Villar & G. Montserrat (JACA 456786). **SPAIN**. **Huesca**: Aneto, crests of Vallibierna, 17 Aug 1984, G. Montserrat (JACA 182884); Benasque, Mulleres peak, 19 Aug 1995, J.V. Ferrández & J.A. Sesé (JACA 237095); Benasque-Vallibierna, Aneto, under the Coronas hill, 29 Aug 1990, D. Gómez & P. Castro (JACA 221890); Gistain, Viados, Pico Machimala, 16 Aug 1990, P. Montserrat (JACA 207090); Panticosa, crests of Batans or Baldairán, Catieras, 8 Jul 1981, M. Arbella & L. Villar (JACA 114381).

Festuca brigantina* subsp. *actiophyta Gutiérrez Villarías. **SPAIN. La Coruña**: between S. André de Teixido y Cariño, 1 Nov 1992, J. Amigo & M.I. Romero (FCO 19848); Sierra de La Capelada, O Bico, 22 Jun 1993, M.I. Gutiérrez Villarías, J. Amigo, M.I. Romero & J. Homet (FCO 19858, FCO 19859, FCO 19860 & FCO 19861); Sierra da La Capelada, O Bico, near S. André de Teixido, 1 Nov 1992, J. Amigo & M.I. Romero (FCO 19847); Ibídem, Vixia Herbeira, 23 Jun 1993, M.I. Gutiérrez Villarías, J. Amigo, M.I. Romero & J. Homet (FCO 19851 & FCO 19853).

Festuca brigantina* subsp. *brigantina (Markgr.-Dann.) Markgr.-Dann. **PORTUGAL**. **Tras-os-Montes**: Bragança, Alimonde, 12 Jun 2010, C. Aedo et al. (MA 823390); near Carracedo, 24 Jun 1966, P. Silva, B. Rainha & J. Martins (MA 286778); Carracedo, Sardoal, Serra da Nogueira, 17 Aug 1995, L.M. Ferrero (MA 787933); crossroads Mosqueira, 6 Jul 1994, S. Castroviejo & C. Aguilar (MA 681255).

Festuca capillifolia Dufour in Roem. & Schult. **SPAIN. Alicante**: Benifato, Sierra de Aitana, Font de Partagat, road to Pas de la Rabosa, 27 May 2014, M.A. Alonso, B. Crespo & G. Martínez (COFC 61531); **Almería**: Sierra de Gádor, Barranco de La Atalaya, 24 May 1995, E. Giménez & F. Gómez Mercado (HUAL 6088); Berja, Sierra de Gádor, Los Morrones from Pozo Lupión, 11 Jun 2014, E. López & G. Martínez (COFC 61573).

Festuca carpetana Fuente, Sánchez-Mata & Rivas Mart. **SPAIN**. **Madrid**: Alto del León, Puerto de Guadarrama, 24 Jun 2015, J.A. Devesa & G. Martínez (COFC 61996 & COFC 62001); Alto de los Leones, track of the Puerto de Guadarrama to Peguerinos, 14 Jun 2014, G. Martínez & C. Morales (COFC 61537); Las Guarramas, Sierra de Guadarrama, 5 Jul 1977, S. Rivas-Martínez (MA 364194); La Morcuera, 25 Jul 1954, without collector (MA 773507); Puerto de Canencia, 24 Jun 2015, J.A. Devesa & G. Martínez (COFC 62003); Puerto de la Morcuera, near the Refugio de la Morcuera, 24 Jun 2015, J.A. Devesa & G. Martínez (COFC 62002); Puerto de Navacerrada, going up to the Alto de las Guarramillas, 14 Jun 2014, G. Martínez & C. Morales (COFC 61538); Ibídem, 24 Jun 2015, J.A. Devesa & G. Martínez (COFC 62138 & COFC 61986); Rascafría, Valcotos, climb to the laguna de Peñalara, 20 Jul 1996, Martín-Blanco (MA 594207).

Festuca clementei Boiss. **SPAIN**. **Granada**: Sierra Nevada, ascension to Veleta peak from the Posiciones, 24 Jul 2013, P. Barberá, J.A. Devesa, A. Díaz, E. León, E. López & G. Martínez (COFC 61232); Ibídem, Monachil, climb to Veleta, 12 Jul 2000, P.

Catalán, J.A. López- Rodríguez, P. Torrecilla (MA 780512); Ibídem, summit of Veleta, 12 Jul 1986, B. Díez Garretas & A. Asensi (MGC 18778).

Festuca frigida (Hack.) K. Richt. **SPAIN**. **Granada**: Capileira, Sierra Nevada, Lagoon of Aguas Verdes, 6 Aug 2003, Díaz & Comino (MGC 54871); Ibídem, 7 Aug 2014, E. López & E. León (COFC 61410); Güejar-Sierra, Sierra Nevada, Corral del Mulhacén, Lagoon of La Mosca, 2 Oct 1975, Fdez. Casas & García Guardia (MA 394491).

Festuca glacialis Miégev. **SPAIN**. **Cantabria**: Hoyos Engros, Los Urrieles, 12 Aug 1986, Moreno (MA 681846); Picos de Europa, Camaleño, Horcados Rojos, 13 Aug 1987, Moreno (MA 681845); **Gerona**: Nuria to Noufonts, 5 Sep 1913, Sennen (MA 12129); **Huesca**: Aisa, Foya de Aragues, 12 Jul 1990, D. Gómez (MA 590631); Ibídem, Lecherín peak, 27 Aug 1985, D. Gómez & P. Montserrat (MA 478505); Monte Perdido, Torla, 7 Aug 1974, P. Montserrat (MA 286733 & MA 363080); Torla, Bujaruelo, 19 Aug 1992, P. Montserrat & Villar (MA 581466); **Palencia**: Velilla of Carrión river, Espigüete, 13 Jul 1995, Nieto Feliner et al. (MA 560276).

Festuca glauca Vill. **SPAIN**. **Gerona**: Cabo de Creus, climb to the Monasterio de Sant Pere de Rodas from Port de la Selva, 21 May 2012, J.A. Devesa & E. López (COFC 61169); La Escala, in the vicinity of the ruins of Ampurias, 22 May 2014, E. López & G. Martínez (COFC 62017).

Festuca gracilior (Hack.) Markgr.-Dann. **SPAIN**. **Barcelona**: Can Jorba, 14 Jun 1985, Nuet Badia & Panareda (BC 675576); **Lérida**: La Noguera, Mont Roig, 18 Jun 1984, Romo (BC 831554); **Tarragona**: Prades, 18 May 1952, Batalla (BC 124717).

Festuca gredensis Fuente & Ortúñez. **SPAIN**. **Ávila**: between Garganta del Villar and Navacepedilla de Corneja, Puerto de Chía, 4 Jul 2012, J.A. Devesa (COFC 61000 & COFC 61003); Plataforma de Gredos, 4 Jul 2012, J.A. Devesa (COFC 61002); Ibídem, 24 Jun 2015, J.A. Devesa & G. Martínez (COFC 62000); Puerto del Pico, 4 Jul 2012, J.A. Devesa, (COFC 61001); Ibídem, 23 Jun 2015, J.A. Devesa & G. Martínez (COFC 61997); **Cáceres**: Tornavacas, Peñanegra towards La Garganta, 27 Jun 2012, E. López & M. López (COFC 60992); **Salamanca**: Candelario, birth of the Cuerpo de Hombre river, near the Refugio, 28 Jun 2012, E. López & M. López (COFC 61105); Ibídem, Sierra de Candelario, La Covatilla Ski Resort, 23 Jun 2015, J.A. Devesa & G. Martínez (COFC 61987 & COFC 62156).

Festuca henriquesii Hack. **PORTUGAL**. **Beira Alta:** Torre, Sierra de la Estrella, between Sabugueiro and Covilha, 22 Jun 2015, J.A. Devesa & G. Martínez (COFC 62030).

Festuca hystrix Boiss. **SPAIN**. **Álava**: Audícana, 17 Jun 1984, P.M. Uribe-Echebarría (MA 318743); **Burgos**: Lastras de las Eras, 10 Jul 2012, E. López & S. Patino (COFC 61121); **Granada**: Puebla de Don Fadrique, Sierra de Guillimona, Puerto de la Losa, 20 Jun 2013, E. López & G. Martínez (COFC 61264); Sierra Nevada, Collado de las Sabinas, 24 Jul 2013, P. Barberá, J.A. Devesa, A. Díaz, E. León, E. López & G. Martínez (COFC 61238 & COFC 61233); on the climb to the Veleta peak, Km-34, Las Sabinas, 9 Jul 2012, J.A. Devesa & M. López (COFC 60988); **Jaén**: Sierra de Cazorla, Campos de Hernán Perea, Refugio de la Monterilla, 3 Jun 2014, E. López & G. Martínez (COFC 62018); Ibídem, Nava Noguera, 11 Jun 2015, E. León & G. Martínez (COFC 62047); Sierra de Mágina, on the road of ascent to Mágina peak from Mata-

Bejid, 24 Jun 2013, J.A. Devesa, E. López & G. Martínez (COFC 61235, COFC 61236 & COFC 61237); Valdepeñas de Jaén, Sierra de la Pandera, on the descent from the military base, 11 Jun 2013, J.A. Devesa, E. López & G. Martínez (COFC 61234); Ibídem, 30 May 2014, E. León & G. Martínez (COFC 61873); **Málaga**: Tolox, Sierra de las Nieves, Cueva del Oso hill, 9 Jul 2014, B. Cabezudo, G. Martínez & F. Soriguer (COFC 61577).

Festuca indigesta Boiss. **SPAIN**. **Almería**: Berja, Sierra de Gádor, base of the Morrones from Pozo Lupión, 11 Jun 2014, E. López & G. Martínez (COFC 61405); **Granada**: road from Guadix to Bayárcal, on the climb to Puerto de La Ragua, 12 Jul 2013, J.A. Devesa & G. Martínez (COFC 61243); Sierra Nevada, Peñones de San Francisco, 9 Jul 2012, J.A. Devesa & M. López (COFC 60983); Ibídem, 24 Jul 2013, P. Barberá, J.A. Devesa, A. Díaz, E. León, E. López & G. Martínez (COFC 61225); Ibídem, on the climb to Veleta peak, Km-34, Las Sabinas, 9 Jul 2012, J.A. Devesa & M. López (COFC 60984); Puerto de la Ragua, 19 Jun 2013, E. López & G. Martínez (COFC 61242).

Festuca liviensis (Verg.) Markgr.-Dann. **ANDORRA. Andorra La Vieja**: 4 Jul 1992, C. Navarro et al. (MA 525958). **SPAIN**. **Gerona**: Planolas, 23 Jul 1967, Losa Quintana, Fernández Casas & Boldú (MA 415909); Sarèje, 26 Jun 1926, F. Sennen (MA 470016); **Lérida**: Sierra del Cadí, 25 Jul 1906, C. Pau (MA 59456); Ibídem, 26 Jul 1906 (MA 59455).

Festuca longiauriculata Fuente, Ortúñez & Ferrero. **SPAIN**. **Almería**: Sierra de los Filabres, Calar Alto, near to observatory, 19 Jun 2013, E. López & G. Martínez (COFC 61278); **Granada**: Puerto de la Ragua, 12 Jul 2013, J.A. Devesa & G. Martínez (COFC 61285); Ibídem, road from Guadix to Bayarcal, 12 Jul 2013, J.A. Devesa & G. Martínez (COFC 61279); Ibídem, towards the pass, 19 Jun 2013, E. López & G. Martínez (COFC 61280); Sierra de Baza, El Raposo, 14 Jun 1984, J. Torres, G. Blanca & C. Morales (GDAC 26308); Ibídem, 13 Jun 1985, J. Torres, G. Blanca & C. Morales (GDAC 26311); Ibídem, Pico Padilla, 20 Jun 1985, J. Torres, G. Blanca & C. Morales (GDAC 26310).

Festuca marginata* subsp. *alopecuroides (Hack.) K. Rich. **FRANCE**. **Pyrénées-Atlantiques**: Lescun, Cayolar d'Anaye, 12 Jul 1998, Patino & Valencia (COFC 62171). **SPAIN**. **Alicante**: Benifato, Font de Forata, 6 Jul 1993, Solanas (ABH 7905); **Gerona**: Maçanet de la Selva, 31 May 1945, Font Quer (BC 868850); Olopte - Tossal d'Isovol, 28 Jun 2006, Romo & Nualart (BC 866340); Vall de Nuria, road to Pico Noufont from the Santuario de Nuria, 23 Apr 2014, E. López & G. Martínez (COFC 61578); **Huesca**: Formigal, 4 Jul 2013, J.A. Devesa & G. Martínez (COFC 61460); **Lérida**: Serra del Cadí, Prat d'Aguiló, 9 Jul 1949, De Bolòs et al. (BC 822761).

Festuca marginata* subsp. *andres-molinae Fuente & Ortúñez. **SPAIN**. **Barcelona**: Montserrat, top of Montgros, 2 May 2003, Pyke & Pallàs (BC 905794); **Tarragona**: Alfara de Carles, NW of Vall Cervera, 17 May 1986, De Torres (BC 905474); Coll del Caragol, 8 Jun 1999, Navarro et al. (MA 626894); Monte Caro, without date, Font Quer (BC 69748); **Zaragoza**: Calatayud, Embid de la Ribera, Peña de la Mora, 29 May 2006, Pyke (BC 905757).

Festuca marginata* subsp. *marginata (Hack.) K. Rich. **ANDORRA**. **La Massana**: Arinsal, 5 Jul 1992, Montserrat & Benito (BC 878917). **FRANCE**. **Ardèche**: Near Mayres, Rocher d'Abraham, 1 Jul 1971, Auquier (MAF 96011); **Drôme**: Romans-sur-Isère, Les Balmes, 28 May 1972, Auquier et al. (MAF 98943); **Meurthe-et-Moselle**: Francheville, N of Toul, 5 May 1973, Auquier (MAF 98942); **Yvelines**: Gommécourt, 9 Jun 1980, De Retz (MAF 122776). **SPAIN**. **Gerona**: Llivia, in the mountain of Llivia Castle, 24 May 2014, E. López & G. Martínez (COFC 62021).

Festuca michaelis Cebolla & Rivas Ponce. **SPAIN**. **Guadalajara**: between Copernal and Espinosa de Henares, 26 May 2013, G. Martínez & C. Morales (COFC 61334); road to Redueña, 28 May 2013, G. Martínez & C. Morales (COFC 61333); **Madrid**: Guadalix de la Sierra, in the abandoned quarry, 28 May 2013, G. Martínez & C. Morales (COFC 61330); Patones, Pontón de la Oliva, 24 May 2013, G. Martínez & C. Morales (COFC 61332); road from Torrelaguna to La Cabrera, 28 May 2013, G. Martínez & C. Morales (COFC 61331).

Festuca niphobia (St.-Yves) Kerguélen. **ANDORRA**. **Ordino**: Coll d´Ordino, 5 Jul 1992, P. Montserrat, D. Gómez & J.L. Benito (JACA 172092); Coll d´Ordino-Casamanya, 5 Jul 1992, P. Montserrat, D. Gómez & J.L. Benito (JACA 175892 & JACA 175992).

Festuca ochroleuca Timb.-Lagr. **SPAIN**. **Barcelona**: Borredà, next the hermitage of Sant Martí de Boatella, 15 Jun 2011, N. Ibáñez et al. (BC 923578); Ibídem. Riera de Merlés, 17 Jun 2015, E. López & G. Martínez (COFC 62087); **Gerona**, Sadernas, road to Sant Aniol d'Aguja, beside riera de Sant Aniol, 23 May 2014, E. López & G. Martínez (COFC 61583); Between San Jaime de Llierca and Santa Magdalena de Montpalau, 22 May 2014, E. López & G. Martínez (COFC 61585).

Festuca plicata Hack. **SPAIN**. **Alicante**: Cocentaina, Summit of Mont Cabrer, 24 Jun 2013, C. Morales (COFC 61240); **Cádiz**: Grazalema, Sierra del Pinar, Cerro San Cristóbal and crest of Pinar, 1 Jul 2008, B. Cabezudo, A.V. Pérez-Latorre, O. Gavira, M. Becerra & F. Soriguer (MGC 68480); **Córdoba**: Sierra de la Horconera, Pico Morrón, 3 Nov 2013, E. León & G. Martínez (COFC 61255); **Granada**: Sierra de Baza, Zújar, Cerro Javalcón, 12 Jul 1971, Fernández Casas (MA 415888).

Festuca querana Litard. **SPAIN**. **Zamora**: Sierra de la Culebra, between Folgoso de la Carballeda and Pedroso de la Carballeda, 1 Jul 2014, E. López, G. Martínez & P. Bariego (COFC 61511).

Festuca reverchonii Hack. **SPAIN**. **Granada**: Huéscar, Sierra de Guillimona, 9 Jun 1997, V. de la Fuente, L.M. Ferrero & E. Carrillo (MAF 159658); Sierra de Castril Natural Park, North sector of the park, 16 Jun 1992, C. Morales & C. Passera (GDAC 37556); **Jaén**: Santiago de la Espada, Calar de las Palomas, 3 Jun 1983, C. Soriano (MA 462161); Sierra de las Banderillas, top of Peña del Águila, 18 Jun 1976, F. Muñoz Garmendia & C. Soriano (MA 462162); Sierra de Cazorla, shelter of Rambla Seca, 3 Jun 2014, E. López & G. Martínez (COFC 61363).

Festuca rivas-martinezii Fuente & Ortúñez. **SPAIN**. **Ávila**: Between Garganta del Villar and Navacepedilla de Corneja, Puerto de Chía, 4 Jul 2012, J.A. Devesa & G.

Martínez (COFC 61504); **Madrid**: Sierra de Guadarrama, Alto de los Leones, 14 Jun 2014, E. López (COFC 61498).

Festuca segimonensis Fuente, Joch. Müll. & Ortúñez. **SPAIN**. **Albacete**: From Yeste to Calar de la Sima, 28 Jun 2016, J.L. Cánovas, J.A. Devesa, J. Guerra, J.F. Jiménez, G. Martínez & P. Sánchez (COFC 62563 & COFC 62564); **Granada**: Sierra de Guillimona, Jul 1907, E. Reverchon (MA 12046); Ibídem, 9 Jun 1997, V. de la Fuente, L.M. Ferrero & E. Carillo (MAF 159667); Sierra de Castril, beside the northern border with Jaén province, 15 Jun 1994, F. Gómez Mercado & J.F. Mota (HUAL 12869); **Jaén**: Santiago de la Espada, 14 Jun 1956, L. Ceballos (MA 170016); Sierra de Castril, Jun 1903, E. Reverchon (MA 265492); Sierra de la Malessa, Jul 1904, E. Reverchon (MA 12045).

Festuca summilusitana Franco & Rocha Afonso. **PORTUGAL**. **Beira Alta:** Manteigas, from Pousada de San Lorenço toward Penhas Doradas, Serra da Estrela, 22 Jun 2015, J.A. Devesa & G. Martínez (COFC 61985 & COFC 61998); Ibídem, Penhas da Saude, Serra da Estrela, Alto dos Livros, 24 Jun 2014, E. López, J. Jansen, F.J. Valtueña & C.G. Relinque (COFC 61544); Ibídem, Tapada Dr. Antonio, 24 Jun 2014, E. López, J. Jansen, F.J. Valtueña & C.G. Relinque (COFC 61609); Sabugueiro, toward Torre, Serra da Estrela, 22 Jun 2015, J.A. Devesa & G. Martínez (COFC 62007); Torre, Serra da Estrela, 22 Jun 2015, J.A. Devesa & G. Martínez (COFC 61999 & COFC 62033); Serra da Estrela, 15 Jun 1982, J. Guerra (MGC 41314); Serra da Estrela, Facarao, Jul 1906, M. Ferreira (MA 265608); Serra da Estrela, Poço do Inferno, 15 Jun 1949, R. Fernandes & Sousa (MA 286685). **SPAIN. León**: Molinaferrera, Bounzamariel Valley, 18 Jul 1947, without leg. (MA 12053)**;** Morales del Arcediano, 14 Jun 1978, F. Llamas (MA 489037, MGC 12589 & MGC 13287); Ponferrada, Montes Aquilianos, near Pico Tesón, 12 Jul 1981, Lansac & Nieto (MA 503786)**;** Sierra del Teleno, 18 Jul 1947 (MA 59411 & MA 59412); Tabladillo, Jul 1946 (MA 12057)**;** Truchas, Sierra de la Cabrera, en el Vizcodillo, 5 Jul 1978, E. Temprano (MA 503788)**;** Villalibre de Somoza, 2 Jul 1980, F. Llamas (MA 448005); **Orense:** Sierra do Invernadeiro, Fraga da Serra da Pena, 23 Jun 1973, S. Castroviejo (MA 197332); Sierra del Invernadeiro, near Pico Seixo, 5 Aug 1989, S. Castroviejo (MA 471663); **Zamora:** Porto, La Fraga, 19 Aug 1991, Aldasoro (MA 585401); Ibídem, reservoir of Cárdena, 19 Aug 1991, Aldasoro (MA 585304).

Festuca valentina (St.-Yves) Markgr.-Dann. **SPAIN**. **Alicante** Cocentaina, Sierra Mariola, Mont Cabrer, 15 Jun 2013, G. Martínez (COFC 61262, COFC 61263, COFC 61265 & COFC 61277); Ibídem, 24 Jun 2013, C. Morales (COFC 61258 & COFC 61259); Ibídem, Font del Povet, 24 Jun 2013, C. Morales (COFC 61261).

Festuca vasconcensis (Markgr.-Dann.) Auquier & Kerguélen. **SPAIN. Asturias:** Oviedo, Cabo de Peñas, 14 Jul 2016, J.A. Devesa & G. Martínez (COFC 62834); **Cantabria:** San Vicente de la Barquera, Oyambre Natural Park, 14 Jul 2016, J.A. Devesa & G. Martínez (COFC 62829).
Festuca vettonica Fuente, Ortúñez & Ferrero. **SPAIN**. **Ávila**: La Serrota, 7 Aug 1983, M. Luceño (MA 508364); Ibídem, Canto de la Oración, Sierra de La Paramera, 22 Jun 1995, V. de la Fuente & L.M. Ferrero (MAF 159682).

Festuca yvesii Sennen & Pau. **ANDORRA**. **Ordino**: Casamanya Peak, 5 Jul 1992, D. Gómez & J.L. Remon (JACA 190592). Ibídem, 17 Aug 1998, E. Ortúñez & R. Gamarra

(JACA 57599); **SPAIN**. **Gerona**: Vallter 2000 Ski Station, Plans de Coma Armada, 9 Jul 1999, E. Ortúñez & R. Gamarra (JACA 57699); **Huesca**: Jaca, Oroel-W, Barranco Fondo, 9 Jul 1986, P. Montserrat (JACA 478986); L´Empriu, Cerler, from Bacive, 5 Aug 1987, P. Monts, L.V. & D.G (JACA 138287); Plan, climb to Puig Alfar from the Collado de las Coronas, 16 Jul 1981, D. Gómez, F. Fillat & G. Montserrat (JACA 877581); **Lérida**: Ruda, 11 Jul 1992, P. Montserrat, J.L. Benito et al. (JACA 246592).

Sect. Aulaxyper Dumort.

Festuca heterophylla* subsp. *braun-blanquetii Fuente, Ortúñez & Ferrero. **SPAIN**. **Lérida**: Vall Ferrera, ravine d' Aixens, 31 Aug 2002, Aedo, Aizpuru & Pedrol (MA 700473); **Navarra**: from Puerto de Belagua to Isaba, 17 Jul 2012, J.A. Devesa & E. López (COFC 61137).

Festuca heterophylla* subsp. *heterophylla Lam. **SPAIN**. **Barcelona**: Torelló to Bellmunt, Jul 1910, Sennen (MA 12260); **Girona**: El Gironès, Aiguaviva, Masrocs river, 28 May 2009, J. Calvo (MA 789647).

Festuca iberica (Hack.) K. Richt. **PORTUGAL**. **Tras-os-Montes**: Alimonde, Serra da Nogueira, 30 Jun 2014, E. López & G. Martínez (COFC 61608). **SPAIN**. **Granada**: Baza Natural Park, Prados del Rey, 19 Jun 2014, E. López & G. Martínez (COFC 61551); **Guadalajara**: Luzaga, Luzaga camp, 26 May 2013, G. Martínez & C. Morales (COFC 61484); road from Cifuentes to Canredondo, 15 May 2013, G. Martínez & C. Morales (COFC 61479); **Jaén**: Sierra de Cazorla, Nava Noguera, 11 Jun 2015, E. León & G. Martínez (COFC 62046); **Zamora**: Moral de Sayago, proximity to the Villalcampo dam, 30 Jun 2014, P. Bariego, E. López & G. Martínez (COFC 61861).

Festuca juncifolia Chaub. **SPAIN**. **Cantabria**: San Vicente de la Barquera, Oyambre Natural Park, 14 Jul 2016, J.A. Devesa & G. Martínez (COFC 62830); **Vizcaya**: Gorliz, Gorliz beach, 9 Jul 2012, E. López & S. Patino (COFC 61117).

Festuca nevadensis (Hack.) K. Richt. **SPAIN**. **Almería**: Sierra de Gádor, surroundings of Pozo Lupión, 11 Jun 2014, E. López & G. Martínez (COFC 61869); **Granada**: Puebla de Don Fadrique, Sierra de Guillimona, Puerto de la Losa, 20 Jun 2013, E. López & G. Martínez (COFC 61308); **Jaén**: Sierra Mágina; ascent to the Pico Mágina from Mata-Bejid, 24 Jun 2013, J.A. Devesa, E. López & G. Martínez (COFC 61424); between Guadix to Bayarcal, Puerto de la Ragua, 12 Jul 2013, J.A. Devesa & G. Martínez (COFC 61315).

Festuca nigrescens Lam. **SPAIN**. **Cantabria**: Santander, Puerto de San Glorio, viewpoint of the Collado de Llesba, 11 Jul 2012, E. López (COFC 61110); **Navarra**: Irati forest, 17 Jul 2012, J.A. Devesa & E. López (COFC 61111).

Festuca pyrenaica Reut. **SPAIN**. **Huesca**: Puerto de Sahún, between Chía and Plan, Sierra de Chía, 25 Jul 2014, E. López & G. Martínez (COFC 61415); Saravillo, Cotiella massif, from Circo de Gallinas to Cotiella, 23 Aug 1978, P y G. Montserrat (BC 636733); **Lérida**: Senet, La Colladeta, 9 Sep 2011, M. Guardiola & A. Petit (BC 877286).

Festuca rivularis Boiss. **SPAIN**. **Granada**: León, lagoon of Aguas Verdes, 7 Aug 2014, E. López & E. León (COFC 61429); **Huesca**: Formigal, 4 Jul 2013, J.A. Devesa

& G. Martínez (COFC 61459); **Soria**: Laguna Negra, 2 Jul 2013, J.A. Devesa & G. Martínez (COFC 61450).

Festuca rothmaleri (Litard.) Markgr.-Dann. **SPAIN**. **Ávila**: Navarredonda, 04 Jul 2012, J.A. Devesa (COFC 61012); **Madrid**: Sierra de Guadarrama, Alto de los Leones, 14 Jun 2014, E. López (COFC 61496); **Zamora**: Ribadelago, Sanabria, 01 Jul 2014, P. Bariego, E. López & G. Martínez (COFC 61602).

Festuca rubra* subsp. *juncea (Hack.) K. Richt. **ANDORRA**. **Encamp**: Envalira pass, road to the Maia peak, 4 May 2014, E. López & G. Martínez (COFC 62394 & COFC 61995).

Festuca rubra* subsp. *pruinosa (Hack.) Piper. **SPAIN**. **Asturias**: Bañugues, near the Cabo de Peñas, 14 Jul 2016, J.A. Devesa & G. Martínez (COFC 62833); **Cantabria**: Islares, 13 Jul 2016, J.A. Devesa & G. Martínez (COFC 62828); **Guipúzcoa**: Fuenterrabia, Higuer lighthouse, 13 Jul 2016, J.A. Devesa & G. Martínez (COFC 62825).

Festuca rubra* subsp. *rubra L. **SPAIN**. **Burgos**: Puerto de Angulo, 10 Jul 2012, E. López & S. Patino (COFC 61108); **Gerona**: Vall de Nuria, road to Noufont peak from the Nuria Sanctuary, 23 Apr 2014, E. López & G. Martínez (COFC 61556); **Navarra**: climb to the Port of Belagua, 3 Jul 2013, J.A. Devesa & G. Martínez (COFC 61456); **Orense**: road of climb to the mountain station of Cabeza de Manzaneda, 17 Jul 2013, E. López & G. Martínez (COFC 61488); **Palencia**: Puerto de Piedrasluengas, 11 Jul 2012, E. López (COFC 61112).

Festuca trichophylla (Gaudin) K. Richt. **SPAIN**. **Bisaurri**: Mount Baciero, Gabás, 4 Jul 1987, G. Montserrat & J.A. Sese (SANT 38198); **Huesca**: climb to the Ermita San Antón de Torla, 4 Jul 2013, J.A. Devesa & G. Martínez (COFC 61445); **Tarragona**: Vallfogona de Riucorb, between Balneario and Segura, 17 Jun 2015, E. López & G. Martínez (COFC 62081).

Sect. Eskia Will

Festuca burnatii St.-Yves. **SPAIN**. **Asturias**: Macizo de Peña Ubiña, 15 Jun 1968, M. Mayor (SEV 24002); **Cantabria:** Santander, Puertos de Aliva, 15 Jul 1976, Casaseca, Ladero & G. López (MA 263193); **León**: El Soto, 15 Jul 1978, Castroviejo, G. López & E. Valdés-Bermejo (MA 547954); Ponferrada, Montes Aquilianos, near Peñalba de Santiago, 19 Jun 1981, Alamillo, Castroviejo, Fdez. Quirós & Nieto (MA 502941); Priaranza del Bierzo, Montes Aquilianos, Ferradillo, 18 Jul 1982, G. Nieto Feliner (MA 317267); Puerto de Cubillos, 13 Jun 1970, J. Andrés (SEV 16729).

Festuca elegans Boiss. **SPAIN**. **Ávila**: Navarredonda de la Sierra, near Navarredonda, 4 Jul 2012, J.A. Devesa (COFC 61007); **Cáceres**: Tornavacas, Peñanegra toward La Garganta, 27 Jun 2012, E. López & M. López (COFC 60993); **Ciudad Real**: Fuencaliente, in the picnic area next to the Camping de San Isidro, 7 Jun 2013, J.A. Devesa, E. López & G. Martínez (COFC 61517); **Granada**: Puerto de la Ragua, 12 Jul 2013, J.A. Devesa & G. Martínez (COFC 61328); **Málaga**: Sierra de las Nieves Natural Park, Parauta, 28 Jun 2012, B. Cabezudo, R. Casimiro-Soriguer, J.A. Devesa & E. Ruiz

de Clavijo (COFC 61008); **Zamora**: Ribadelago, Sanabria Lake, 1 Jul 2014, P. Bariego, E. López & G. Martínez (COFC 61607).

Festuca eskia Ramond ex DC. **ANDORRA**. **Encamp**: Puerto de Envalira, road to the Pic de Maia, 24 May 2014, E. López & G. Martínez (COFC 62016). **FRANCE.** Bielsa tunnel, 20 Jul 2016, J.A. Devesa & G. Martínez (COFC 63331). **SPAIN. Cantabria**: Las Segas peak, Peña Sagra mountain range, Lamasón, 3 Aug 1985, C. Aedo (MA 623669); **Huesca:** Astún, Truchas tarn, 21 Jul 2016, J.A. Devesa & G. Martínez (COFC 63334); **Palencia**: Fuentes Carrionas National Reserve, from Cardaño de Arriba to Peña Prieta, Natural Spring of Las Lomas, 22 Aug 1998, C. Dobeš & E. Vitek (MA 624583).

Festuca gautieri (Hack.) K. Richt. **SPAIN**. **Albacete**: Nerpio, Sierra de las Cabras, Southwest slope of the Macalón, 7 Jul 2001, J.M. Herranz & M.J. Martínez Lirola (MA 697087); **Alicante**: Carrascal de Alcoy, 7 Aug 1958, A. Rigual (MA 372937); **Cuenca**: Tajo river, upstream, 8 Jul 2008, L.M. Ferrero, A. Hamplová, J. M. Herranz, O. Mayoral, L. Medina, C. Soriano & A. Vela (MA 782935); **Gerona**: Cadí mountain range, pr. Arseguel, 23 Jul 1993, C. Aedo et al. (MA 529426); **Guipúzcoa**: Oñate, Sierra de Aizkorri, Artzamburu, 06 Jul 1985, J.A. Alejandre (MA 339954); **Jaén**: Sierra de Cazorla, Pico Cabañas, 6 Aug 1968, A. Segura Zubizarreta (MA 509156); **Navarra:** Coll de la Pierre de Saint Martín, on the border with Francia, 19 Jul 2016, J.A. Devesa & G. Martínez (COFC 63332); Isaba – France, Roncalia ski station, 19 Jul 2016, J.A. Devesa & G. Martínez (COFC 63333).

Festuca* × *picoeuropeana Nava. **SPAIN**. **Asturias**: Collado de la Fragua, on Vega Redonda, Picos de Europa, 8 Dec 1983, C. Aedo (MA 623698); **Huesca**: Canfranc, Candanchú, El Tobazo, 21 Jul 1996, P. Catalán & V. Mirones (MA 780495); Ordesa National Park, Sierra Custodia, 11 Jun 1997, P. Catalán, V. Mirones (MA 780494); **Lérida**: Las Colladinas, 22 Jun 1996, P. Catalán & V. Mirones (MA 780496).

Sect. Subbulbosae Nyman ex Hack.

Festuca baetica (Hack.) K. Richt. **SPAIN**. **Granada**: Sierra Nevada, Peñones de San Francisco, 9 Jul 2012, J.A Devesa & M. López (COFC 61186); **Jaén**: Sierra Mágina, downhill road from Almadén peak, 29 Jun 2013, J.A. Devesa, E. López & G. Martínez (COFC 61347); **Málaga**: Sierra de las Nieves Natural Park, Parauta, 28 Jun 2012, B. Cabezudo, R. Casimiro-Soriguer, J.A. Devesa & E. Ruiz de Clavijo (COFC 60990).

Festuca durandoi Clauson. ***F. durandoi* subsp. *capillifolia*** (Pau ex Willk.) Rivas Ponce, Cebolla & M. B. Crespo. **SPAIN**. **Castellón**: Espadán peak, 16 Jun 1988, C. Cebolla & M.A. Rivas Ponce (COFC 61596); **Madrid**: Puerto de Somosierra, 12 Aug 1992, prados, C. Cebolla & M.A. Rivas Ponce (COFC 61597).

Festuca paniculata (L.) Schinz & Thell. sl. **SPAIN**. **Burgos**: Espinosa de los Monteros, Puerto de Lunada, 10 Jul 2012, E. López & S. Patino (COFC 61116); **Guadalajara**: Luzaga, 26 May 2013, G. Martínez & C. Morales (COFC 61348); between Cifuentes to Canredondo, 25 May 2013, G. Martínez & C. Morales (COFC 61346); **Huesca**: Tarns of the Ayanet, 27 Jul 1992, C. Cebolla, López Rodríguez & M.A. Rivas Ponce (COFC 61599); **Salamanca**: Candelario, birth of the Cuerpo de Hombre river, near Refugio, 28 Jun 2012, E. López & M. López (COFC 61107); **Zamora**: Ribadelago, Sanabria lake, 1 Jul 2014, P. Bariego, E. López & G. Martínez (COFC 61548).

Sect. Lojaconoa Catalán & Joch. Müll.

Festuca coerulescens Desf. **SPAIN**. **Cádiz**: Algeciras, Sierra de la Palma, 29 Apr 1991, Cebolla & Rivas Ponce (MA 529675); San Roque, Apr 1965, Borja, Mansanet & Monasterio (MA 181357); **Málaga**: Cortes de la Frontera, Las Alegrías farmhouse, 30 Apr 1983, A. Aparicio & S. Silvestre (MA 461551).

Festuca patula Desf. **SPAIN**. **Ciudad Real**: Fuencaliente, 7 Jun 2013, J.A. Devesa, E. López & G. Martínez (COFC 61407); **Córdoba**: Cabra, La Nava de Cabra, 19 Jun 2012, E. Triano, J.A. Devesa & E. López (COFC 61163); Hornachuelos, Guadalora river, 13 May 1993, R. Pinilla & R. Tamajón (COFC 22715); **Málaga**: Sierra de las Nieves Natural Park, Los Quejigales, 28 Jun 2012, B. Cabezudo, R. Casimiro-Soriguer, J.A. Devesa & E. Ruiz de Clavijo (COFC 60998).

Sect. Scariosae Hack.

Festuca scariosa (Lag.) Asch. & Graebn. **SPAIN**. **Almería**: Berja, Sierra de Gádor, next to Pozo Lupión, 11 Jun 2014, E. López & G. Martínez (COFC 61565); **Córdoba**: Cabra, Picacho de Cabra, 19 Jun 2012, J.A. Devesa, E. Triano & E. López (COFC 61166); **Granada**: road from Guadix to Bayarcal, climb to Port of La Ragua, 12 Jul 2013, J.A. Devesa & G. Martínez (COFC 61326); Trevenque, Road from La Cortijuela farmhouse, 25 Jul 2013, P. Barberá, J.A. Devesa, A. Díaz, E. León, E. López & G. Martínez (COFC 61228); **Jaén**: Sierra de la Pandera, 11 Jun 2013, J.A. Devesa, E. López & G. Martínez (COFC 61426).

Sect. Pseudoscariosa Krivot.

Festuca pseudeskia Boiss. **SPAIN**. **Granada**: Sierra Nevada, Peñones de San Francisco, beside the university hostel, 9 Jul 2012, J.A. Devesa & M. López (COFC 60985); Ibídem, Peñones de San Francisco, 24 Jul 2013, P. Barberá, J.A. Devesa, A. Díaz, E. León, E. López & G. Martínez (COFC 61226).

B. SUBG. DRYMANTHELE Krecz. & Bobr.

Sect. Phaeochloa Griseb.

Festuca altissima All. **SPAIN**. **Cantabria**: Mount Robea, pr. Ledantes, Vega de Liébana, 13 Aug 1988, Carlos Aedo (MA 615670); **Lugo**: Seoane de Caurel, Devesa da Rogueira, 19 Jul 1989, S. Castroviejo, B. Casaseca & E. Rico (MA 483565).

Festuca lasto Boiss. **SPAIN**. **Málaga**: Birth of the Hoyo del Bote river, 28 Jun 2012, B. Cabezudo, R. Casimiro-Soriguer, J.A. Devesa & E. Ruiz de Clavijo (COFC 61011).

C. SUBG. SCHEDONORUS (P. Beauv.) Peterm.

Sect. Schedonorus (P. Beauv.) W.D.J. Koch

Festuca arundinacea Schreb. **SPAIN**. **Gerona**: road from Arbucíes to Sant Hilari, 21 May 2012, J.A. Devesa & E. López (COFC 61172); **Huesca**: Nuestra Señora de Torla,

4 Jul 2013, J.A. Devesa & G. Martínez (COFC 61340); **Navarra**: between Erro and Roncesvalles, 16 Jul 2012, J.A. Devesa & E. López (COFC 61125).

Festuca interrupta Desf. **SPAIN**. **Alicante**: Cocentaina, climb to Mont Cabrer, Sierra Mariola, 15 Jun 2013, G. Martínez (COFC 61354); **Gerona**: between Port-Bou and Colera, 20 Aug 1981, J. Molero (COFC 52427).

Festuca mediterranea (Hack.) Rouy ex Prain
SPAIN. **Málaga**: Sierra de las Nieves Natural Park, Los Quejigales, 28 Jun 2012, B. Cabezudo, R. Casimiro-Soriguer, J.A. Devesa & E. Ruiz de Clavijo (COFC 60994).

Sect. Plantynia (Dumort.) Tzvelev

Festuca gigantea (L.) Vill. **SPAIN**. **Gerona**: Queralb, Freser river, 9 Aug 1985, J.A. Mejías, J. M. Polo & C. Romero (SEV 125668); **Huesca**: Pineta-La Larry, 6 Aug 1977, P. Montserrat (SEV 51705).
